# Flying Chameleons: A New Concept for Minimum-Deployment, Multiple-Target Tracking Drones

**DOI:** 10.3390/s22062359

**Published:** 2022-03-18

**Authors:** Manuel Vargas, Carlos Vivas, Francisco R. Rubio, Manuel G. Ortega

**Affiliations:** 1 Department of Automation and Systems Engineering, University of Seville, Camino de los Descubrimientos s/n, 41092 Seville, Spain; vivas@us.es (C.V.); rubio@us.es (F.R.R.); mortega@us.es (M.G.O.); 2 Laboratory of Engineering for Energy and Environmental Sustainability, University of Seville, Camino de los Descubrimientos s/n, 41092 Seville, Spain

**Keywords:** multi-camera tracking, aerial surveillance, autonomous agent, optimization strategies

## Abstract

In this paper, we aim to open up new perspectives in the field of autonomous aerial surveillance and target tracking systems, by exploring an alternative that, surprisingly, and to the best of the authors’ knowledge, has not been addressed in that context by the research community thus far. It can be summarized by the following two questions. Under the scope of such applications, what are the implications and possibilities offered by mounting several steerable cameras onboard of each aerial agent? Second, how can optimization algorithms benefit from this new framework, in their attempt to provide more efficient and cost-effective solutions on these areas? The paper presents the idea as an additional degree of freedom to be exploited, which can enable more efficient alternatives in the deployment of such applications. As an initial approach, the problem of the optimal positioning with respect to a set of targets of one single agent, equipped with several onboard tracking cameras with different or variable focal lengths, is addressed. As a consequence of this allowed heterogeneity in focal lengths, the notion of distance needs to be adapted into a notion of *optical range*, as the agent can trade longer Euclidean distances for correspondingly longer focal lengths. Moreover, the proposed optimization indices try to balance, in an optimal way, the verticality of the viewpoints along with the optical range to the targets. Under these premises, several positioning strategies are proposed and comparatively evaluated.

## 1. Introduction

In the last decade, the general public has become increasingly familiar with the concept of combining a remotely-piloted or unmanned aerial vehicle (UAV) with an actuated electromechanical device, mounting a camera as a compound gadget that is suitable for a myriad of applications that require putting “eyes up on the sky”, ranging from military purposes to entertainment activities [1].

In the most elementary case, this electromechanical device, called a gimbal, is a simple stabilizer for the camera’s point of view, compensating for the eventual orientation changes derived from the vehicle’s own movement, attempting to fixate a preset inertial orientation for the camera.

In the second step, the gimbal has a dual function, as a stabilizer and tracking device. In this case, apart from compensating for the tilts and/or turns undergone by the vehicle, it is desired to simultaneously keep track of moving targets [2,3,4,5,6]. The introduction of zoom camera lenses brought superior mission capabilities [7,8]. Having high magnification factors, however, makes fine image stabilization a serious challenge, usually requiring the conjunction of high-precision gimbal control and optical or electronic image stabilization strategies [9,10].

When the objective is to track, conceivably with such a level of visual detail, not one but several targets that may be distant from each other, existing works can be categorized into two possibilities. The first and obvious one consists of deploying several vehicles with similar capabilities. The second, on the other hand, focuses on shared attention, where a single vehicle periodically switches the aiming direction of its camera among several targets, so as not to permanently lose sight of any of them.

The use of multiple aerial vehicles for detection, surveillance, and tracking of multiple targets has largely been covered in the literature [11,12]. Fewer studies have been devoted to the second category, where a shared-attention strategy for a single aerial vehicle or a coordinated team is addressed [13,14,15]. This second category is nevertheless quite appealing, aiming to accomplish the task with minimum resource deployment while making the most out of the available resources. This is highly advantageous, not only in terms of the economic cost of physical deployment, but also in regard to energy requirements during mission execution. At the same time, it is an approach minimally intrusive with the environment where the mission is to be performed, which can be very relevant in certain contexts (concealed or covert military operations, wildlife monitoring, etc.). On the other hand, it has the obvious handicap that each of the targets can only be monitored during a fraction of time inversely proportional to the number of targets, taking into account the possible “blind” transition intervals, when no target may be in the field of view of the switching camera. Even though this strategy may be sufficient in tracking applications with a reduced number of targets with predictable trajectories, if these conditions are not met, the observation rate may become a compromising factor. Moreover, even if this intermittence might be acceptable for target location estimation and prediction, in applications where the observation or filming of the activity of individual targets is of critical interest, it may be highly undesirable to have intervals during which such activity remains completely ignored.

In addition to these two options, namely, the increase in the number of engaged UAVs or the use of some shared-attention strategy, already studied and exploited to date, this paper studies a third option that has not been previously explored and which, in the opinion of the authors, deserves serious consideration today, given the inexorable reduction in costs and simultaneous increase in the computational power of onboard systems. The idea is to equip one single vehicle with several gimbal-camera sets, in order to enable the continuous tracking of targets with the desired level of visual detail, while preserving the advantages of the minimal-deployment paradigm. The lower the ratio gimbal-camera set cost/UAV cost, the more attractive this option becomes. Each one of these vehicles or agents is referred to, in the title of this work, as a *flying chameleon,* for obvious reasons.

On the other hand, this new alternative does not exclude the possibility of multiple agents with this multi-camera configuration, if the number of targets or the mission so justifies. It enables the introduction of an additional dimension or a new degree of freedom to address the problem not considered so far. In the context of multi-target tracking systems, this new dimension can, therefore, be viewed as an extension of the two pre-existing alternatives already discussed. For example, when the number of targets and the potential separation between them, in relation to the desired level of visual detail, are sufficiently high, a solution that encompasses all three approaches can be considered; that is, multiple vehicles, each one equipped with several steerable cameras, all combined with a shared-attention strategy. Additionally, by contributing toward minimizing the number of vehicles involved in a given surveillance or target-tracking mission, this proposal helps to reduce costs, energy consumption, and simplifying collision avoidance planning.

However, this third approach entails a new problem associated with the optimal positioning/trajectory specification for the vehicle, in order to accomplish the surveillance or tracking tasks with the desired level of quality. In this regard, there are some aspects of the problem, which, despite being necessary, can be considered “solved issues” from a theoretical and technological point of view, such as target aiming with steerable cameras or UAV control and standard trajectory tracking. This work, however, is focused on developing a novel conceptual and theoretical framework, taking that suggested third option as a new paradigm, with application to multi-target surveillance and tracking systems and enabling previously unattainable optimal solutions for agent positioning.

The structure of the paper is as follows. Section 2 describes the related state-of-the-art and outlines the main contributions of the work. Section 3 formally introduces the proposed framework and describes the computations involved in target geolocation and target aiming. Section 4 proposes several alternatives for optimization indices, looking for the ideal positioning of a single multi-camera agent with respect to the set of targets of interest. Section 5 presents a set of simulation experiments that demonstrate the feasibility of the proposed approach and compare the performance of the different strategies to address it. Finally, the paper ends with the conclusions of the study and some comments referring to future lines of work.

## 2. State-of-the-Art and the Main Contributions

To the best of the authors’ knowledge, there are no previous works covering the idea of autonomous surveillance or tracking of multiple targets by means of several steerable onboard cameras on each aerial vehicle. However, there are previous works where multiple cameras with fixed orientations were mounted on the same UAV, but none in the context of multi-target tracking, where, as we will see, the ability to freely aim each camera to a desired direction introduces very interesting possibilities. All known previous multi-camera setups, such as [16,17,18], were intended to stereo, multi-view fusion, electro-optical-thermal imaging combination, visual odometry, or photogrammetry. According to this, the present section is devoted toward describing areas that are more closely related to this work from a scientific point of view or “escenarios” that are more relevant to the applications of the concepts here discussed.

### 2.1. One UAV, Single or Multiple Targets

Relevant existing works devoted toward the surveillance or tracking of one or several targets are restricted to the use of a single aerial agent equipped with a steerable or fixed camera. Choi and Kim [3] presented a target tracking algorithm for a fixed-wing unmanned aerial vehicle equipped with a gimbaled monocular-vision sensor. The system was intended to track another single air vehicle. The range observability problem that arises from the combination of a monocular sensor and target not being constrained, to move on a single plane, is discussed and solved via a nonlinear adaptive observer, allowing the estimation of the target states. They also developed a guidance law for the UAV. Additionally, maneuvers for guaranteeing persistent excitation, which is related to the convergence of the adaptive observer, are proposed. In the work of Farmani et al. [4], one single UAV equipped with a gimbaled camera is used to track and geolocate multiple mobile ground targets. A set of extended Kalman filters are used to estimate each target’s state (ground-plane position and velocity). The highest target-density region is identified, and a model predictive control strategy is used to estimate the camera pose minimizing the overall uncertainty in the target state estimates. In [5] the authors make use of a fixed-wing UAV with an onboard gimbaled thermal camera, in order to detect and track floating objects in high seas environments, particularly emphasizing the problem of real-time georeferencing of the target. The authors of [6] propose strategies to localize, recognize, and track targets using images acquired by a single agent. In this case, the acquired information is integrated with a geographic information system, as a means of improving the target geolocation and motion prediction and providing contextual information. Additional important aspects addressed in that work are image stabilization, intelligent auto focus, and low-resolution image-based target detection and recognition.

### 2.2. Multiple UAVs, Single or Multiple Targets

The problem has also been escalated by the introduction of a team of UAVs for the tracking of a single or multiple targets. Zhang and Liu [19] presented a strategy for multiple UAVs tracking a single uncooperative target. The bottom layer of the approach implements a sliding mode control-based loitering algorithm for each individual fixed-wing UAV by simply regulating the distance to the target, while the top layer enables formation flight cooperative target tracking by the set of UAVs. Morbidi and Mariottini [20] described an active single-target tracking strategy to deploy a team of UAVs, equipped with three-dimensional range-finding sensors, along paths that minimize the uncertainty of the target’s position. Sung et al. [21] proposed a distributed approach for the allocation problem in a multi-robot–multi-target tracking context, with the objective of dealing with both limited sensing and communication capabilities.

The problem grows in complexity when the team of UAVs have to simultaneously keep track of several moving targets. Two interesting surveys on this matter can be found in [11,12]. In this context, it is also worth mentioning the work from Toupet and How [22], where they propose a collaborative optimal resource allocation method for multiple UAVs to distribute their locations over an area of interest, while performing multiple target tracking. Resource allocation is formulated as an optimal control problem through mixed-integer linear programming. The objective of this optimal formulation is to maximize the cumulative tracking performance over all targets, allowing more than one UAV to track each single target, as it is shown that the cumulative tracking accuracy is thus improved. Adamey and Ozguner [23] also consider the problem of cooperative multi-target tracking and surveillance using multiple UAVs. They develop a decentralized particle filter approach for target location estimation and a strategy for agent mobility management via dynamic region allocation among the UAVs.

In this same category, it is also of interest to mention the more recent works by Farmani et al. [24] and Sung et al. [21]. In [24], the authors extend their previous development to a system of multiple UAVs, developing a scalable, cooperatively decentralized, multi-target localization, and tracking system that minimizes the overall uncertainty of the estimated target locations. The proposal is structured in three steps. First, extended Kalman filters are used for the estimation of each individual target state and combined with a DBSCAN clustering strategy to group targets, based on their estimated relative distance on the ground plane. In a second step, an optimal sensor manager is introduced, based on directed weighted graphs and predictive control, in order to allocate clusters to vehicles and to determine the high-density points inside each cluster, where the cameras have to be aimed to. Finally, an optimal distributed path planner is added, intended to minimize a combination of three aspects, namely, the distance to the targets, the uncertainty in the target geolocation, and the rate of change of this uncertainty. Sung et al. [21], on the other hand, proposed a distributed approach for the allocation problem in a multi-robot–multi-target tracking context, with the objective of dealing with both limited sensing and communication capabilities.

### 2.3. Visual Coverage

In addition to the previous works, there is a parallel line of research devoted to the so-called visual-coverage problem, where tasks, such as surveillance or tracking, are commonly formulated as an optimization problem that aims to maximize the coverage of a set of targets or an interest area. For instance, in [1], the authors propose strategies to control the position and orientation of onboard cameras, in order to achieve equal visual coverage of the ground plane. In [25], coverage strategies with a team of aerial vehicles are also proposed, but using fixed downward facing cameras instead. The purpose in this case is planning trajectories to track as many targets as possible, while estimating their locations. The problem is formulated as a maximum group coverage problem, following two variants: maximizing the number of targets being tracked subject to a desired tracking quality per target, or maximizing the overall tracking quality. More recently, the coverage problem has also been addressed from a resilience point of view in [26], where is it assumed that a fraction of the drones can eventually suffer malfunction or be under attack. Persistent coverage control for a team of UAVs featuring inertially-fixed, downward-facing cameras is addressed by Hatanaka et al. in [27]. The authors make use of the concept of control barrier functions, in order to ascertain the satisfaction of diverse constraints, such as the coverage condition, collision avoidance, or preservation of a minimum battery level.

### 2.4. Shared Attention

In an attempt to maximize the use of the available resources, strategies based on shared attention have also been proposed in the literature. Sharma and Pack [13] implemented the shared-attention strategy for a team of small fixed wing UAVs by a model predictive control based cooperative sensor resource manager, which is in charge of the geolocalization and tracking of multiple moving ground targets. More recently, Baek and York [15] presented a decentralized framework for multiple UAVs, allowing them to effectively manage their sensors to detect and locate multiple mobile targets. A formulation based on the unscented Kalman filter is used to optimally estimate and predict the location of the ground targets, while the allocation of targets is continuously updated among the cooperating agents using consensus decision-making. Zhao et al. [14] also addressed the cooperative decision-making problem for multi-target tracking when using a team of UAVs. In this case, the tracking decisions are made under the framework of distributed multi-agent partially-observable Markov decision processes.

Many of these strategies can be revisited under the perspective of having at our disposal several independently steerable onboard cameras on each vehicle.

### 2.5. Contributions of the Work and Main Areas of Application

The more relevant contributions of the paper are outlined next. To begin with, this work introduces a new proposal for an autonomous multi-target, multi-camera, aerial tracking system, which, to the best of the authors’ knowledge, has not been previously studied. From the theoretical point of view, the proposal is kept generic, assuming an unspecified and possibly arbitrary large number of tracking cameras. The interest of such a proposal has already been highlighted in the previous section.

Some particularly suitable areas of application of the proposed framework can be identified. High seas search and rescue missions are a good example [28,29,30], where people and/or small boats adrift at sea after shipwrecks or other disasters need to be tracked as closely and continuously as possible, in order to assess the prioritization strategy for rescue teams. Another example is surveillance missions involving suspicious vessels [31]. It is common that, for instance, smugglers or illegal migration mafias coordinate the simultaneous departure of several vessels, including even decoy vessels, making it harder to detect or track all of them. In such a context, the proposed strategy could significantly contribute to the gathering of visual evidence against criminal acts or reinforce the border control mechanisms [32,33,34,35]. Covert military operations have been already mentioned, but homeland security missions, securing areas, convoy protection [36], etc., could also benefit from this approach; in sporting events [37,38], where the goal is to simultaneously follow several targets of interest, while minimizing intrusion in the scene; cinematographic and audiovisual works [39,40], with the intent of simultaneously shooting several points of view from the same trajectory in the air; wildlife monitoring, attempting to study animal behavior in relatively open areas [41,42], with minimal intrusion into the environment; and in traffic surveillance applications, enabling simultaneous tracking and close-up filming of the behavior of several vehicles [43,44].

A second contribution of the work is the description of a first theoretical approach to the problem, where the flat-earth assumption is made, allowing a simple solution to the concurrent problem of relative 3D location of targets, in order to reconstruct their trajectories. In our context, it is assumed that no stereo or multi-view reconstruction techniques can be applied, since the different camera views can be, in general, utterly not overlapping. Under the previous assumptions, the solution for the geometry involved in the initial target allocation and target position reconstruction is described.

Finally, as the most relevant contribution of the work, the problem of the autonomous optimal positioning of a single agent is addressed, coming up with performance functions adapted to the new framework, looking for suitable trade-offs between distance to targets and verticality of the points of view. The convenience of the combination of such factors, compared to other alternatives that can be adopted from the literature, is also discussed.

## 3. Framework

In this section, the description of the proposed concept is given first. Then, the geometry of the problem is described under the flat-earth assumption, which avoids the need of stereo, elevation maps, or any other range-gathering mechanism.

### 3.1. Working Scenario

**Figure 1:**
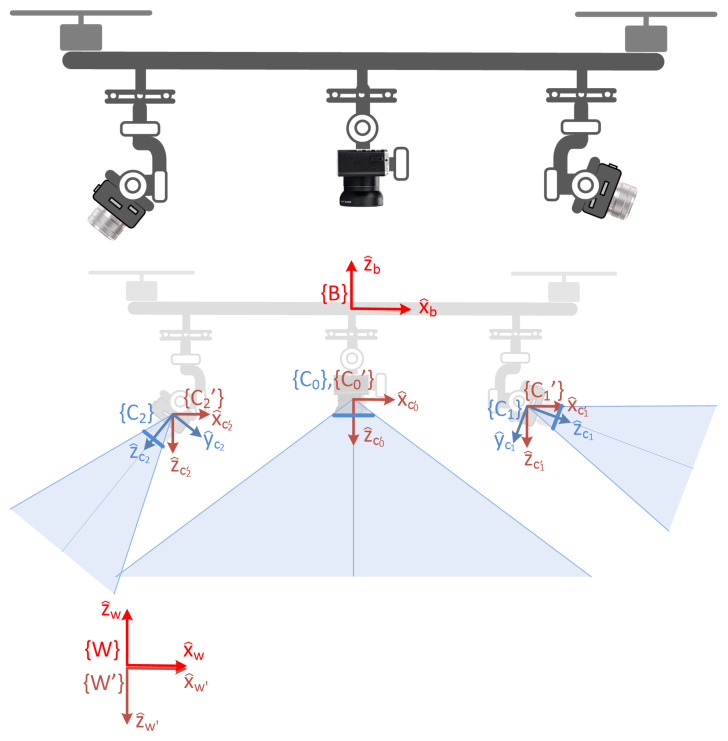
Illustration of the concept using a multirotor platform, a central wide-angle camera on a 2-DOF gimbal, and two tracking cameras with their respective 3-DOF gimbals.

The concept can be implemented in quite diverse aerial platforms, ranging from light multirotor systems to heavy fixed wing autonomous drones. The proposed onboard multi-camera setup is composed of an optional wide-angle central camera, denoted as $\left\{C_{0}\right\}$, flanked by *n* tracking cameras, $\left\{C_{i}\right\}\;\forall \;i\;=\;\left\{1\ldots n\right\}$. While a simpler 2-DOF gimbal could suffice for the central camera (provided that it is admissible that the camera yaw angle be “locked” to the main body yaw angle of the vehicle), the purpose of this central camera gimbal is to ensure that a perfect cenital point-of-view for this camera is preserved at all times, regardless of the vehicle’s maneuvers, full 3-DOF gimbals are required, in general, for each tracking camera. In Figure 1, this concept is sketched for a particular case of a multirotor vehicle and n=2.

If present, the central camera would preferably be a wide or ultra-wide angle camera, in order to perceive the whole scene below, presumably with low resolution. The tracking cameras, on the other hand, will ideally be equipped with motorized zoom lenses, allowing imaging more distant targets with sufficient resolution.

As a case of study, the descriptions given in the rest of the document will be particularized for a holonomic agent, such as a quadcopter system, with a central wide-angle camera and an unspecified number of tracking cameras. Figure 1 shows the involved coordinate frames. First, the world reference frame {W}, for which a configuration *East–North–Up (ENU)* is assumed, being located at a convenient stationary position on the terrain surface. For convenience, an inverted version of this reference system, called $\left\{W^{\prime }\right\}$, is also introduced, obtained from {W} after a $180^{\circ }$ rotation around the ${\hat{x}}_{w}$ axis. Another reference frame, denoted as {B}, is rigidly attached to the vehicle platform. Two different frames are defined for each one of the cameras. $\left\{C_{i}\right\}$ is the actual camera frame, according to the instantaneous orientation provided by the associated gimbal. $\left\{C_{i}^{\prime }\right\}$, having its origin coincident with that of $\left\{C_{i}\right\}$, has otherwise a fixed orientation, in particular, the same as $\left\{W^{\prime }\right\}$:

$${}^{w^{\prime }\;}R_{c_{i}^{\prime }}=I_{3}\;,\;\forall \;i\in \left\{0,\ldots n\right\}$$

where $I_{3}$ is the identity matrix.

For the central camera, since a perfectly zenithal point of view is generally desired, it is expected that the only possible difference of $\left\{C_{0}\right\}$ with respect to $\left\{C_{0}^{\prime }\right\}$ is the yaw angle; that is, a single rotation around the ${\hat{z}}_{c_{0}^{\prime }}$ axis.

In our context, multi-target tracking has two levels. At the bottom level, regardless of where the flying platform is positioned, the problem of target aiming must be solved. At the top level, assuming that the targets are being correctly tracked, a steering strategy must be designed so that the vehicle can be optimally positioned with respect to the targets, according to a given criterion.

#### General Assumptions and Simplifications

Some a priori conditions are assumed in the following description. First, the fact that the flat-earth assumption holds. This simplification is approximately valid in some contexts, having been exploited by many other authors, as in [4,5,13,15,24,25,31].

Secondly, it is assumed that an estimate of the vehicle’s altitude and global position are available at all times, given by the vehicle’s onboard navigation sensors. Additionally, it is assumed that, as usual, each gimbal–camera assembly is equipped with a dedicated IMU providing the inertial orientation of the corresponding camera. These “secondary” IMUs are independent of the main IMU mounted in the main body of the vehicle and essential for the UAV’s attitude control. Specifically, the information provided by *i*-th IMU is the relative orientation of $\left\{C_{i}\right\}$ with respect $\left\{C_{i}^{\prime }\right\}$. In the absence of this secondary IMUs, the instantaneous information from the main IMU has to be combined with the gimbal encoder readings, in order to retrieve the required inertial orientation.

In order to simplify the subsequent geometric description, it is also assumed that the camera is mounted on the corresponding gimbal in such a way that any rotation induced by the gimbal occurs around the camera’s nodal point (origin of $\left\{C_{i}^{\prime }\right\}$ in our depiction), so no parallax effect will be appreciated. As the cameras are usually mounted, in practice, in a way that minimizes the inertia induced by gravity, it is hardly the case. However, assuming that the distance from the theoretical nodal point to the center of gravity is much shorter than the distances to the objects of interest, this approximation will prevail. In any case, the visual-feedback gimbal control loop will take charge of any misalignment during real-time target aiming.

Without loss of generality, in our description, the effect of lens distortion will be disregarded. It is assumed that, in the presence of significant lens distortion, the images undergo a rectification process before being processed.

In this initial approach, the description is limited to the one-to-one scenario, where the number of targets does not exceed the number of tracking cameras. Future work will cover the study of clustering strategies to deal with more general scenarios. On the other hand, despite the existence of zooming effect in the tracking cameras being a very interesting feature, in this initial approach, focal lengths are not formulated as optimization variables that can be automatically adjusted in real time, according to a given criterion. Instead, despite some of these focal lengths might vary from time to time, their values are introduced as parameters into the optimization problem. In accordance with this, it will be assumed that all tracking cameras have known, but in general different, focal lengths.

Finally, the descriptions of any low-level attitude control of the aerial platform or the low-level gimbal control are out of the scope of the paper, as they are issues largely solved even by manufacturers.

### 3.2. Camera Model

The adopted model describing the projection of relevant scene points onto the images is described here. A standard pin-hole camera model is assumed for each camera. The list of parameters is the following:

- $\omega_{s}$: sensor width [m].
- $h_{s}$: sensor height [m].
- $nPix_{w}$: number of pixels in horizontal direction [pix].
- $nPix_{h}$: number of pixels in vertical direction [pix].
- *f*: focal length [m].
- *s*: skew factor.
- ${\left[u_{0},\;v_{0}\right]}^{\;⊤}$: coordinates of the sensor central point [pix].
- $\rho_{w}$: effective pixel width: $\rho_{w}:=\frac{\omega_{s}}{nPix_{w}}\;\left[m/pix\right]$.
- $\rho_{h}$: effective pixel height: $\rho_{h}:=\frac{h_{s}}{nPix_{h}}\;\left[m/pix\right]$.
- $f_{\;w}$: effective focal length (horizontal) $f_{\;w}:=\frac{f}{\rho_{w}}\;\left[pix\right]$.
- $f_{\;h}$: effective focal length (vertical) $f_{\;h}:=\frac{f}{\rho_{h}}\;\left[pix\right]$.

All of these parameters are assumed to be known from an off-line standard camera calibration procedure. After these parameters, the intrinsic camera matrix can be built:

$$A\;:=\;\left[\begin{array}{ccc} f_{\;w} & s\;f_{\;w} & u_{0}\\ 0 & f_{\;h} & v_{0}\\ 0 & 0 & 1 \end{array}\right]$$

This matrix transforms three-dimensional points in the scene, once expressed with respect to frame $\left\{C_{i}\right\}$, to homogeneous coordinates of the corresponding point in the image. Finally, the 2D pixel coordinates of such points can be obtained:

(1) $$\left[\begin{array}{c} u_{i}^{\prime }\\ v_{i}^{\prime }\\ w_{i}^{\prime } \end{array}\right]\;=\;A_{i}\cdot {}^{c_{i}}p_{t_{i}}\;\;⟹\;\;\left[\begin{array}{c} u_{i}\\ v_{i} \end{array}\right]=\left[\begin{array}{c} \frac{u_{i}^{\prime }}{w_{i}^{\prime }}\\ \frac{v_{i}^{\prime }}{w_{i}^{\prime }} \end{array}\right]$$

where ${}^{c_{i}}p_{t_{i}}$ is the position of the defining point of the *i*-th target, with respect to the corresponding tracking camera $\left\{C_{i}\right\}$.

### 3.3. Gimbal Structure

**Figure 2:**
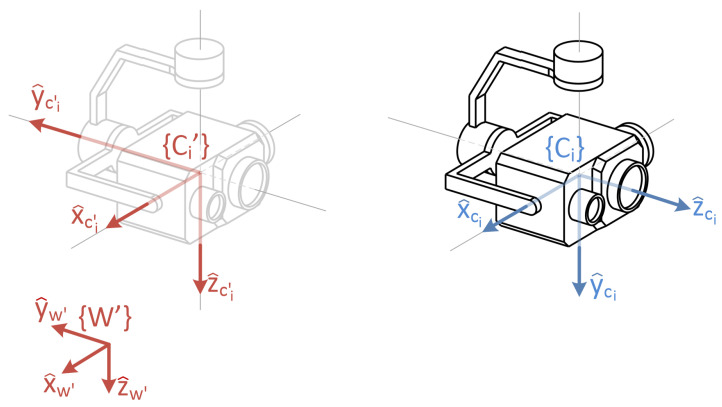
Proposed gimbal structure and associated reference frames.

Figure 2 shows the structure of the 3-DOF gimbals assumed in this work. The location and orientation of the involved reference frames are also depicted.

The origin of both coordinate systems, $\left\{C_{i}\right\}$ and $\left\{C_{i}^{\prime }\right\}$, is set at the camera’s nodal point. As already mentioned, every camera rotation induced by the gimbal is assumed to be made around this very point. In particular, it is assumed that, for each camera, when all three angles are set to zero, the camera is pointing downwards in such a way that $\left\{C_{i}\right\}$ and $\left\{C_{i}^{\prime }\right\}$ are coincident. From this default orientation, the sequence of rotations that allow to set the orientation of $\left\{C_{i}\right\}$ at will is as follows: first, the *yaw* angle (ψ), implies a rotation around ${\hat{z}}_{c_{i}^{\prime }}$ axis. Next, the *roll* angle (ϕ), enables a rotation around new $\hat{y}$ axis. Finally, the *pitch* angle (γ), is a rotation around the resulting $\hat{x}$ axis. The rotation matrix from $\left\{C_{i}^{\prime }\right\}$ y $\left\{C_{i}\right\}$ can be obtained as follows:

$${}^{c_{i}^{\prime }}R_{c_{i}}\;=\;Rot_{z}\left(\psi_{c_{i}}\right)\;\;Rot_{y}\left(\phi_{c_{i}}\right)\;\;Rot_{x}\left(\gamma_{c_{i}}\right)$$

Under this configuration, the *gimbal lock* singularity occurs at $\phi_{c_{i}}=\pm \frac{\pi }{2}\;$[rad]. It is expected that, for the intended applications, where, in general, a steadily horizontal skyline is desired ($\phi_{c_{i}}=0$), the gimbal operation will be always far from this singularity. It is also not expected that the vehicle performs such aggressive maneuvers that force the gimbal, in order to maintain the horizon line with the desired orientation, to drive its second joint close to these limits (it is customary that, in conventional, non-acrobatic multi-rotor vehicles, the reference roll and pitch angles entering the attitude-control system are saturated inside an interval such as [−60,60][deg] or narrower).

In the case of the central camera, a 2-DOF structure would suffice to preserve a zenithal viewpoint, regardless of the vehicle’s maneuvers. In particular, the first rotation (ψ) is the one to be omitted. This entails the limitation that the yaw of the central camera will be forced by the yaw of the vehicle itself.

### 3.4. Target Geolocation

Once a camera has its target in view, whether or not it is in the center of the image, this section describes the geometric relationships allowing the estimation of the global coordinates of that target. That is, how to estimate the full three-dimensional position of the target with respect to {W} from the coordinates of the center point of the target in the image.

With regard to target position estimation and aiming, we are only interested in the position of a specific point of each target, such as the apparent center of its silhouette in the image, regardless of the target orientation. In other words, from the geometrical point of view, the problem can be considered equivalent to the tracking of point-like targets.

#### 3.4.1. Geolocation Using Central Camera

**Figure 3:**
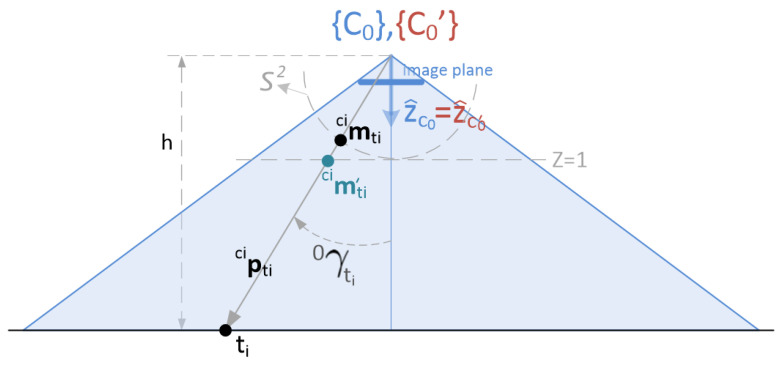
Relation altitude-subtended angle of the projected target point.

First of all, let us consider how to solve the problem of estimating the 3D coordinates of a target in camera $\left\{C_{0}\right\}$. Let us assume that the *i*-th target is observed by the central camera. Denoting by ${\left[{}^{0}u_{i},\;{}^{0}v_{i}\right]}^{\;⊤}$ the coordinates of the relevant point or this target in the image $\left\{C_{0}\right\}$, the geometry involved in the 3D reconstruction of such a point can be observed in Figure 3.

From expression (1), the back projected point is written as:

$${}^{c_{0}\;}p_{t_{i}}\;\propto \;{}^{c_{0}\;}m_{t_{i}}^{\prime }\;:=\;A_{0}^{-1}\cdot \left[\begin{array}{c} {}^{0}u_{i}\\ {}^{0}v_{i}\\ 1 \end{array}\right]$$

It can be seen right away that the third component of ${}^{c_{0}}\;m_{t_{i}}^{\prime }$ is always equal to 1, given the structure of matrix A. That is:

$${}^{c_{0}\;}{m_{t_{i}}^{\prime }}^{⊤}\;e_{3}=1$$

being $e_{3}={\left[0,0,1\right]}^{\;⊤}$. In a similar way, the following will be used later on: $e_{1}={\left[1,0,0\right]}^{\;⊤}$ y $e_{2}={\left[0,1,0\right]}^{\;⊤}$. The vector resulting from the back projection can be normalized into a unitary vector:

$${}^{c_{0}\;}m_{t_{i}}\;:=\;\frac{{}^{c_{0}\;}m_{t_{i}}^{\prime }}{{∥}^{c_{0}\;}m_{t_{i}}^{\prime }∥}\;\equiv \;\frac{{}^{c_{0}\;}p_{t_{i}}}{{∥}^{c_{0}\;}p_{t_{i}}∥}$$

As shown in the figure, the resulting point belongs to the unit sphere, $S^{2}$.

The subtended angle ${}^{0}\gamma_{t_{i}}$ defines the verticality of the target as seen by the central camera. Since the z-axis of this central camera is expected to be always pointing in a perfectly vertical direction, this angle can be obtained as follows:

(2) $$\cos{}^{0}\gamma_{t_{i}}=e_{3}^{\;⊤}\;{}^{c_{0}\;}m_{t_{i}}$$

Hence, by using ${}^{c_{0}\;}m_{t_{i}}$, ${}^{c_{0}\;}p_{t_{i}}$ can be computed:

(3) $${}^{c_{0}\;}p_{t_{i}}\;=\;\frac{h}{e_{3}^{\;⊤}\;{}^{c_{0}\;}m_{t_{i}}}\;\;{}^{c_{0}\;}m_{t_{i}}$$

where we have made use of the fact that the norm of ${}^{c_{0}}\;p_{t_{i}}$ is equal to:

$${∥}^{c_{0}\;}p_{t_{i}}∥\;=\;\frac{h}{\cos{}^{0}\gamma_{t_{i}}}$$

Alternatively, it can be obtained in a more direct way, using ${}^{c_{0}\;}m_{t_{i}}^{\prime }$:

(4) $${}^{c_{0}\;}p_{t_{i}}\;=\;h\;\;{}^{c_{0}\;}m_{t_{i}}^{\prime }$$

From this, the global coordinates of the target can be derived, assuming a known vehicle’s pose:

$${}^{w}\;p_{t_{i}}\;=\;{}^{w}\;p_{c_{0}}+{}^{w}\;R_{c_{0}}{\;}^{c_{0}\;}p_{t_{i}}$$

where:

$${}^{w}\;R_{c_{0}}={}^{w}\;R_{c_{0}^{\prime }}\;{}^{c_{0}^{\prime }\;}R_{c_{0}}\;,\;{}^{w}\;R_{c_{0}^{\prime }}\;=\;{}^{w}\;R_{w^{\prime }}\;=\;Rot_{x}\left(\pi \right)\;,\;{}^{c_{0}^{\prime }\;}R_{c_{0}}\;=\;Rot_{z}\left(-\psi_{b}\right)$$

and $\psi_{b}$ is the vehicle’s yaw angle (positive according to the direction of axis ${\hat{z}}_{b}$, which is opposite to ${\hat{z}}_{c_{0}^{\prime }}$).

#### 3.4.2. Geolocation Using Tracking Cameras

Suppose the *i*-th target is observed by the corresponding tracking camera $\left\{C_{i}\right\}$ (without loss of generality, each target is allotted a number that matches the number of the tracking camera to which it is assigned). The position of the target to be obtained, ${}^{w}\;p_{t_{i}}$, will be computed from the image coordinates in camera $\left\{C_{i}\right\}$: ${\left[u_{i},v_{i}\right]}^{\;⊤}$, from the inertial camera orientation, ${}^{w}\;R_{c_{i}}$, and from the altitude of the vehicle, *h*. Starting from the relation:

(5) $${}^{w}\;p_{t_{i}}\;=\;{}^{w}\;p_{c_{i}}+{}^{w}\;R_{c_{i}}{\;}^{c_{i}\;}p_{t_{i}}$$

where ${}^{c_{i}\;}p_{t_{i}}$ can be obtained from the image point coordinates:

$${}^{c_{i}\;}p_{t_{i}}\;\propto \;{}^{c_{i}\;}m_{t_{i}}^{\prime }:=A_{i}^{-1}\cdot \left[\begin{array}{c} u_{i}\\ v_{i}\\ 1 \end{array}\right]$$

$${}^{c_{i}\;}m_{t_{i}}:=\frac{{}^{c_{i}\;}m_{t_{i}}^{\prime }}{{∥}^{c_{i}\;}m_{t_{i}}^{\prime }∥}\;\equiv \;\frac{{}^{c_{i}\;}p_{t_{i}}}{{∥}^{c_{i}\;}p_{t_{i}}∥}$$

${}^{c_{i}}\;m_{t_{i}}\in S^{2}$ again belongs to the unit sphere, providing the direction of the projection line from the 3D point to the image. This vector is directly linked to the vehicle’s altitude. For the sake of simplicity in this description, the altitude difference between the vehicle’s body center (where the altitude sensor is assumed to be located) and the origin of $\left\{C_{i}\right\}$ is neglected. That is to say, the following approximation is used:

$$h\;=\;{}^{w}\;p_{b}^{\;⊤}\cdot e_{3}\approx {}^{w}\;p_{c_{i}}^{\;⊤}\cdot e_{3}\;\forall i=0\ldots n$$

This does not imply any loss of generality, since the altitude correction can always be made, based on the knowledge of the kinematics of the system.

Matrix ${}^{w}R_{c_{i}}$ can be decomposed as:

$${}^{w}\;R_{c_{i}}\;=\;{}^{w}\;R_{c_{i}^{\prime }}\;{}^{c_{i}^{\prime }}\;R_{c_{i}}$$

${}^{w}\;R_{c_{i}^{\prime }}\;=\;{}^{w}\;R_{w^{\prime }}\;=\;Rot_{x}\left(\pi \right)$, whereas ${}^{c_{i}^{\prime }}\;R_{c_{i}}$ is given by the auxiliary IMU coupled with each tracking camera.

Denoting by $r_{i_{j}}\;\left(j=1\ldots 3\right)$ each column in ${}^{c_{i}}\;R_{c_{i}^{\prime }}$:

$${}^{c_{i}}\;R_{c_{i}^{\prime }}=\left[\begin{array}{ccc} r_{i_{1}} & r_{i_{2}} & r_{i_{3}} \end{array}\right]\;⟹\;{}^{c_{i}^{\prime }}\;R_{c_{i}}\;=\;\left[\begin{array}{c} r_{i_{1}}^{\;⊤}\\ r_{i_{2}}^{\;⊤}\\ r_{i_{3}}^{\;⊤} \end{array}\right]$$

Denoting by $r_{i_{j}}^{\prime }\;\left(j=1\ldots 3\right)$, each one of the columns in ${}^{c_{i}^{\prime }}\;R_{c_{i}}$:

$${}^{c_{i}^{\prime }}\;R_{c_{i}}=\left[\begin{array}{ccc} r_{i_{1}}^{\prime } & r_{i_{2}}^{\prime } & r_{i_{3}}^{\prime } \end{array}\right]\;⟹\;{}^{c_{i}}\;R_{c_{i}^{\prime }}\;=\;\left[\begin{array}{c} r_{i_{1}}^{\prime ⊤}\\ r_{i_{2}}^{\prime ⊤}\\ r_{i_{3}}^{\prime ⊤} \end{array}\right]$$

**Figure 4:**
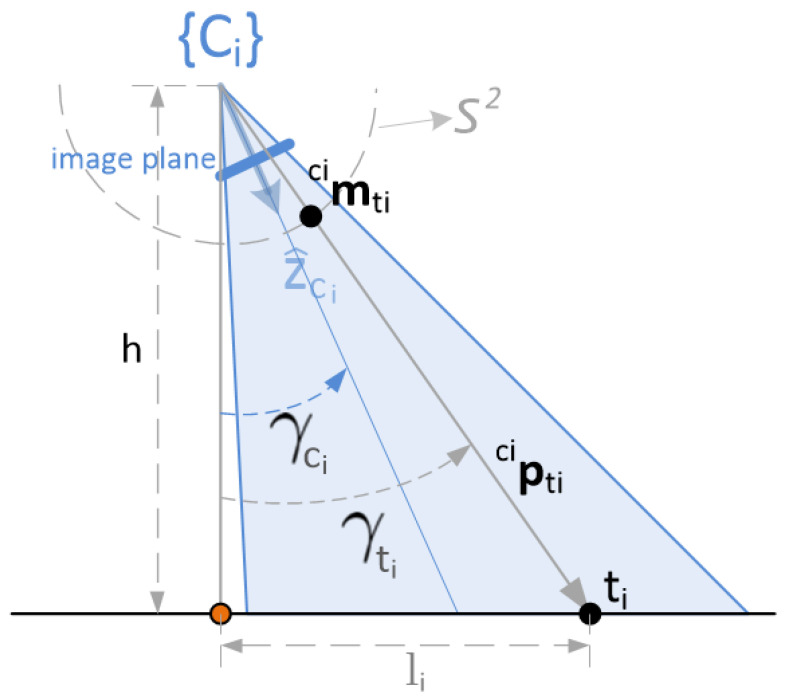
Relation altitude–verticality of the projected target point.

Figure 4 shows the projection of the characteristic target point on the unit sphere and the relation with the altitude.

The angle $\gamma_{t_{i}}$ defines the verticality of the target viewpoint as seen from the camera. On the other hand, $\gamma_{c_{i}}$ describes the verticality of the camera’s aiming direction (verticality of axis ${\hat{z}}_{c_{i}}$), coincident with the inertial angle attained by gimbal’s third joint. Moreover, according to the intended applications, it can be assumed that both angles verify the following condition: $-\pi /2\leq \gamma_{c_{i}},\;\gamma_{t_{i}}\leq \pi /2$.

The angle $\gamma_{t_{i}}$ is clearly related to the distance to the 3D point:

$${∥}^{c_{i}}\;p_{t_{i}}∥\;=\;\frac{h}{\cos\gamma_{t_{i}}}$$

Similar to expression (2), this angle can be linked to the corresponding vector ${}^{c_{i}}m_{t_{i}}$ as follows:

(6) $$\cos\gamma_{t_{i}}\;=\;{}^{c_{i}^{\prime }}m_{ti}^{\;⊤}\;\;e_{3}\;=\;e_{3}^{\;⊤}\;\;\left({}^{c_{i}^{\prime }}\;R_{c_{i}}\;{}^{c_{i}}m_{t_{i}}\right)\;=\;r_{i_{3}}^{\;⊤}{\;}^{c_{i}}m_{t_{i}}$$

With this, the three-dimensional position of the point with respect to the camera, ${}^{c_{i}\;}p_{t_{i}}$, can be reconstructed using the tracking camera, just as it was done with the central camera in (3):

$${}^{c_{i}}\;p_{t_{i}}\;=\;\frac{h}{r_{i_{3}}^{\;⊤}{\;}^{c_{i}}m_{t_{i}}}{}^{c_{i}}m_{t_{i}}$$

Back to the expression (5), the target position with respect to {W} can be obtained:

(7) $${}^{w}\;p_{t_{i}}\;=\;{}^{w}\;p_{c_{i}}+\frac{h}{r_{i_{3}}^{\;⊤}{\;}^{c_{i}}m_{t_{i}}}\;{}^{w}\;R_{c_{i}}{\;}^{c_{i}}\;m_{t_{i}}$$

Equivalently, but just as a function of ${}^{c_{i}^{\prime }}m_{t_{i}}$:

$${}^{w}\;p_{t_{i}}\;=\;{}^{w}\;p_{c_{i}}+\frac{h}{e_{3}^{\;⊤}{\;}^{c_{i}^{\prime }}m_{t_{i}}}\;{}^{w\;}R_{c_{i}^{\prime }}\;{}^{c_{i}^{\prime }}m_{t_{i}}$$

where, as indicated above, ${}^{w}\;R_{c_{i}^{\prime }}$ is a constant matrix: ${}^{w}\;R_{c_{i}^{\prime }}\;=\;Rot_{x}\left(\pi \right)$.

### 3.5. Target Aiming

**Figure 5:**
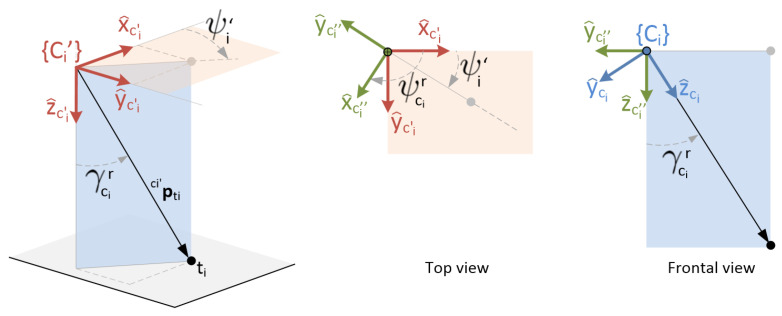
Rotation steps for correct aiming to a target.

Figure 5 illustrates the steps needed for a correct initial pointing to the target of interest, assuming that such a target is already in the field of view of the tracking camera itself.

After the position of the target in the image, the direction of the corresponding 3D projection line from the target can be reconstructed, as described in Section 3.4.2:

$${}^{c_{i}}\;p_{t_{i}}\;\propto \;{}^{c_{i}}\;m_{t_{i}}^{\prime }:=A_{i}^{-1}\cdot \left[\begin{array}{c} u_{i}\\ v_{i}\\ 1 \end{array}\right]$$

$${}^{c_{i}}\;m_{t_{i}}:=\frac{{}^{c_{i}}\;m_{t_{i}}^{\prime }}{{∥}^{c}\;m_{t_{i}}^{\prime }∥}\;\equiv \;\frac{{}^{c_{i}}\;p_{t_{i}}}{{∥}^{c_{i}}\;p_{t_{i}}∥}$$

Knowing the current inertial orientation of the camera $\left\{C_{i}\right\}$, given by ${}^{c_{i}^{\prime }}\;R_{c_{i}}$, we can write:

$${}^{c_{i}^{\prime }}m_{t_{i}}\;=\;{}^{c_{i}^{\prime }}\;R_{c_{i}}{\;}^{c_{i}}m_{t_{i}}$$

The idea is to obtain a new desired reference orientation matrix ${}^{c_{i}^{\prime }}R_{c_{i}}^{r}$, which achieves the correct target pointing.

The rotation necessary to achieve this aiming is performed in two steps. First, a rotation with respect to the ${\hat{z}}_{c_{i}^{\prime }}$ axis to align the *y*-axis with the projection of ${}^{c_{i}^{\prime }}m_{t_{i}}$ onto the plane $\left({\hat{x}}_{c_{i}^{\prime }},{\hat{y}}_{c_{i}^{\prime }}\right)$, but in the opposite direction. Hence, the first rotation corresponds to:

$$Rot_{z}\left(\psi_{c_{i}}^{r}\right)\;,\;\psi_{c_{i}}^{r}\;=\;\frac{\pi }{2}+\psi_{c_{i}}^{\prime }$$

In the central image of the figure, a top view is shown, in which this rotation has been performed, resulting in the intermediate reference frame $\left\{C_{i}^{\prime \prime }\right\}$, in green. The angle $\psi_{c_{i}}^{\prime }$ is obtained from the direction of the unit vector ${}^{c_{i}^{\prime }}m_{t_{i}}$ projected on the plane $\left({\hat{x}}_{c_{i}^{\prime }},{\hat{y}}_{c_{i}^{\prime }}\right)$:

$$\psi_{c_{i}}^{\prime }\;=\;\mathrm{atan}\left(\frac{e_{2}^{\;⊤}\;{}^{c_{i}^{\prime }}m_{t_{i}}}{e_{1}^{\;⊤}\;{}^{c_{i}^{\prime }}m_{t_{i}}}\right)$$

which means that $\psi_{c_{i}}^{r}$ is:

$$\psi_{c_{i}}^{r}\;=\;\mathrm{atan}\left(\frac{e_{1}^{\;⊤}\;{}^{c_{i}^{\prime }}m_{t_{i}}}{-e_{2}^{\;⊤}\;{}^{c_{i}^{\prime }}m_{t_{i}}}\right)$$

According to the assumed configuration of the gimbal structure, the second step could be a rotation with respect to the ${\hat{y}}_{c_{i}^{\prime \prime }}$ axis. However, this inertial *roll* angle would generally be desired to be kept at zero, so that the horizon line coincides with the direction of the horizontal axis of the camera, for the convenience of human supervision. That is, the inertial *roll* angle to be maintained is:

$$\phi_{c_{i}}^{r}=0$$

Thus, the second step is directly the final *pitching* rotation with respect to the ${\hat{x}}_{c_{i}^{\prime \prime }}$ axis, an angle $\gamma_{c_{i}}^{r}$, to get the resulting ${\hat{z}}_{c_{i}}$ axis to point in the direction of the target. This is shown in the front view of the plane $\left({\hat{y}}_{c_{i}^{\prime \prime }},{\hat{z}}_{c_{i}^{\prime \prime }}\right)$, shown on the right side of Figure 5. This second turn will therefore be:

$$Rot_{x}\left(\gamma_{c_{i}}^{r}\right)\;,\;\gamma_{c_{i}}^{r}\;=\;\mathrm{acos}\;\left(e_{3}^{\;⊤}\;{}^{c_{i}^{\prime }}m_{t_{i}}\right)$$

It is assumed—as previously stated—that $\gamma_{c_{i}}$ and $\gamma_{c_{i}}^{r}\in \left(-\frac{\pi }{2},\frac{\pi }{2}\right)$, so there is no ambiguity with the error operation.

In summary, the three inertial angles that must be imposed as a reference to the camera to achieve a correct aiming to the corresponding target are:

(8) $$\begin{array}{ccc} Yaw:\; & \psi_{c_{i}}^{r} & =\mathrm{atan}\left(\frac{e_{1}^{\;⊤}\;{}^{c_{i}^{\prime }}m_{t_{i}}}{-e_{2}^{\;⊤}\;{}^{c_{i}^{\prime }}m_{t_{i}}}\right) \end{array}$$

(9) $$\begin{array}{ccc} Roll:\; & \phi_{c_{i}}^{r} & =0 \end{array}$$

(10) $$\begin{array}{ccc} Pitch:\; & \gamma_{c_{i}}^{r} & =\mathrm{acos}\;(e_{3}^{\;⊤}\;{}^{c_{i}^{\prime }}m_{t_{i}}) \end{array}$$

The corresponding rotation matrix is as follows:

$${}^{c_{i}^{\prime }}\;R_{c_{i}}^{r}\;=\;Rot_{z}\left(\psi_{c_{i}}^{r}\right)\;\;Rot_{x}\left(\gamma_{c_{i}}^{r}\right)$$

### 3.6. Initialization

As already mentioned, the inclusion of the central camera is optional, but one of the aspects that makes it interesting is the simplification of the initialization process. With a simple glance at this image, an operator can manually select the targets of interest, which are then instantly assigned to the tracking cameras. It can also make the system more robust in the event a tracking camera momentarily loses its target. An alternative is that, when the system is started, an algorithm analyzes the wide-angle image and, according to predefined criteria, automatically selects the targets to be tracked.

After this, the initialization process involves two important steps. First, the allocation of targets to tracking cameras and the initial coarse aiming. The latter must ensure that the designated target will be properly centered in the corresponding camera view, close to the center of the image. Following these two initial steps, the visual feedback involved in the ”autonomous” target-aiming control, carried out by each gimbal-camera set, takes charge of continuously regulating the projection of the moving target into the center of the image.

Assuming the one-to-one scenario, one tracking camera has to be allocated to each target. The proposed initial allocation policy is based on a verticality-maximization criterion (the concepts related to this maximum verticality principle will be extensively discussed later on). That is, the combination that maximizes the global verticality of the viewpoints has to be chosen.

Next, we describe the operations required to solve for the initial aiming of the tracking cameras, knowing the position of the target in the wide-angle image and the relative pose between its reference frame, $\left\{C_{0}\right\}$, and the corresponding frame $\left\{C_{i}^{\prime }\right\}\;\forall i=\left\{1,\;\ldots \;n\right\}$. Unlike pointing from the information projected on the camera itself, as just described, pointing from the points projected on the central camera does, in general, require the full three-dimensional coordinates of the point and not just its direction. This will be so unless it can be assumed that the position of the origin of the reference systems $\left\{C_{0}\right\}$ and $\left\{C_{i}\right\}$ are coincident, in which case, there would be no difference from what has just been described in Section 3.5.

As seen in Section 3.4.1, from the projection of the target onto the central camera and the altitude estimate, ${}^{c_{0}}p_{t_{i}}$ can be calculated. Knowing the relative pose between the *i*-th camera and the central camera, ${}^{c_{0}^{\prime }}p_{c_{i}^{\prime }}$, one can write:

$${}^{c_{0}^{\prime }}p_{t_{i}}\;=\;{}^{c_{0}^{\prime }}p_{c_{i}^{\prime }}+{}^{c_{i}^{\prime }}p_{t_{i}}$$

It can be recalled that the orientation of the reference frame $\left\{C_{i}^{\prime }\right\}$ is always the same and coincident with that of the reference frame $\left\{W^{\prime }\right\}$ and that the origin of $\left\{C_{i}\right\}$ is always coincident with that of the corresponding $\left\{C_{i}^{\prime }\right\}$.

Then, from the value of ${}^{c_{0}}p_{t_{i}}$ obtained by the expression (3) or (4), we obtain:

(11) $${}^{c_{i}^{\prime }}p_{t_{i}}\;=\;{}^{c_{0}^{\prime }}R_{c_{0}}{\;}^{c_{0}}p_{t_{i}}-{}^{c_{0}^{\prime }}p_{c_{i}^{\prime }}$$

This is the vector that defines the ideal initial pointing that should be given to the camera $\left\{C_{i}\right\}$, setting the desired direction for the axis ${\hat{z}}_{c_{i}}$, with respect to the reference system $\left\{C_{i}^{\prime }\right\}$. From the unit vector in the target direction, obtained from the three-dimensional point:

$${}^{c_{i}^{\prime }}m_{t_{i}}\;=\;\frac{{}^{c_{i}^{\prime }}p_{t_{i}}}{{∥}^{c_{i}^{\prime }}p_{t_{i}}∥}$$

It is now a matter of applying the same steps seen in expressions (8)–(10) to calculate the two angles to rotate.

## 4. Methods: Multi-Target Optimal Positioning

The problem of the autonomous optimal positioning of the aerial platform, with respect to the set of targets, is formally addressed in this section, ”coming up” with novel optimization indices, as we place special emphasis on the observation of the targets. According to this, the objective might be the optimal positioning of the agent, not only attempting to keep it as close as possible to all of the targets, as conventionally proposed, but doing so while promoting the maximum verticality of the viewpoint, with respect to each one of them. These are two conditions that, to some extent, are in opposition of each other, since reducing the altitude is expected to reduce the distance to the targets, but this will be detrimental to the verticality with respect to them, which, conversely, will increase with the altitude. A similar conflicting situation was faced in [25], but in a different context, where a team of UAVs with single fixed downward facing cameras attempted to track as many targets as possible, while estimating their locations. The increase in altitude allowed more targets in the field of view, at the expense of precision in the location estimation.

Setting a high verticality condition might be of particular interest for several reasons:

- Attempting to minimize variations in the appearance of the target. If no verticality constraints are set, this would leave total freedom to achieve both fully zenithal and fully horizontal views of the target. This would result in a potentially high variability in the target’s appearance, which would make the task of the tracking algorithms more difficult. It is expected that, by maintaining as vertical a view of each target as possible, the variability in the target appearance will be as low as possible.
- Attempting to minimize the occlusions. Given the nature of the proposed problem, in which several targets of unspecified height are moving around, it is clear that giving the possibility to track such targets without minimum verticality and altitude constrains, may lead to situations of more likely occlusions of the targets by other objects, between the targets themselves, or even that one of the onboard cameras gets in the line of sight of another one.
- In applications where it is specifically desired to monitor the targets with minimal intrusion and even where such monitoring should go unnoticed by the targets themselves, it is again interesting to establish a minimum altitude threshold, combined with an optimization of the verticality with respect to the set of targets.

As for the altitude specifications, an altitude range will be set in which the vehicle must be maintained within at all times:

$$0<h_{\min}\leq h\leq h_{\max}$$

The study of the problem incorporating these limits does not lessen its generality, since such limits can be set as loose or strict as desired, provided they are finite. This aspect will be integrated into the optimization problem itself, formulating it as a constrained optimization problem.

First, a couple of strategies merely based on intuitive foundations will be suggested. They will be taken as baseline strategies against which to compare with those that are more mathematically grounded, based on optimization indexes, to be proposed later.

### 4.1. Baseline Target Tracking: Naive Heuristic Strategies

In order to intuitively decide an appropriate location for the autonomous vehicle, it is necessary to consider, on the one hand, the position on the terrain that represents, in some sort, a “center” of the set of targets being tracked and, on the other hand, the altitude at which the vehicle has to be placed, precisely above the chosen central ground position. The difference between the two baseline pursuing strategies to be described is precisely the choice of such a representative center point.

#### Uniform Versus Periphery-Biased Baseline Strategies

The first (and most obvious) choice is to give all targets the same relevance. In this case, the central point will be simply the center of mass of the set of points where the targets are located. This strategy looks to achieve an acceptable point of view, on average, given that all targets are treated uniformly. However, it could lead to situations with very unfavorable points of view for targets further away from the center of mass.

**Figure 6:**
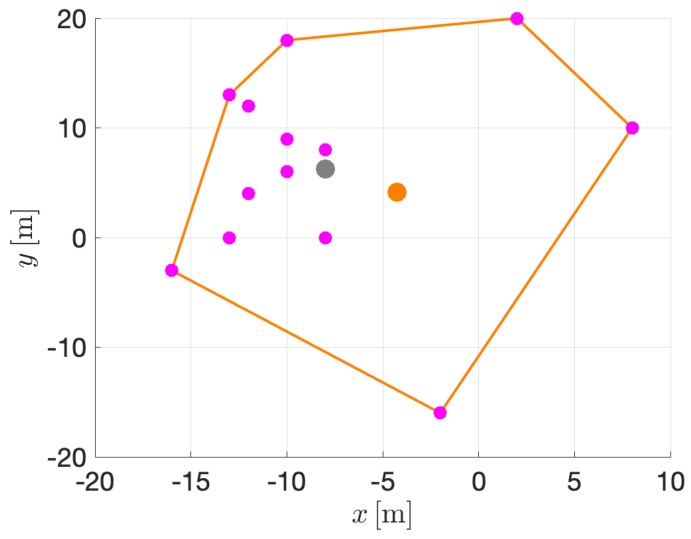
Central point of a cloud of targets by estimation of the convex-hull center (in orange) and by estimation of the center of mass (in gray).

In order to guarantee a better balance in the viewpoints, even for the most peripheral targets, it is possible to propose an alternative in which special relevance is given to such targets. In particular, this can be achieved by choosing as a central point the center of the convex hull of the set. In Figure 6, a zenithal view of a set of targets is sketched. The targets are shown in magenta color, while the polygon corresponding to the convex hull of the set and its center are shown in orange. As can be seen in this case, in order to obtain a more balanced view for all targets, including the outermost ones, the center of the convex hull is preferable to the mere center of mass.

**Figure 7:**
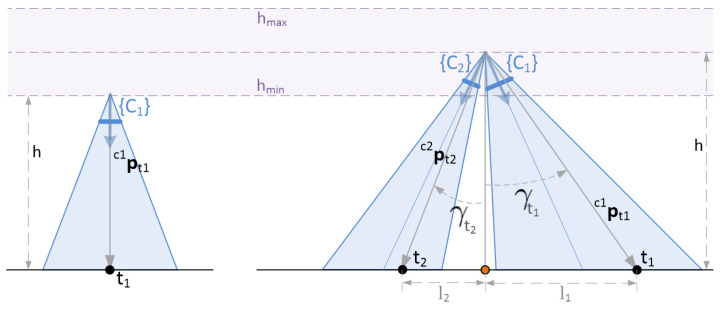
Sketching of two trivial cases. **Left:** one target. **Right:** two targets.

Given a set of targets and once determined at each instant the central point above which the vehicle has to be driven to, it is not straightforward to choose the right altitude, which represents a good proximity/verticality trade-off for the set of targets. Once the center point is fixed, for each particular target, the verticality depends on the altitude as follows: $\gamma_{t_{i}}=\mathrm{atan}\frac{l_{i}}{h}$, where $l_{i}$ is the distance on the plane from the center point to the particular target. This is shown in Figure 7, where the orange spot represents the center point of the target set (again, in this figure, for the sake of simplicity in the description, the distance between the origin of the body reference frame {B} and that of each tracking camera $\left\{C_{i}\right\}$ is assumed to be negligible compared to the distances to the targets).

Once the central point on the ground is chosen, the adoption of an altitude value equal to the minimum, $h=h_{\min}$, will ensure maximum proximity to the targets. On the other hand, the adoption of the maximum allowed altitude, $h=h_{\max}$, will maximize verticality. For this reason and in the absence of any better a priori criterion, in both baseline strategies, it can be decided to set the desired constant altitude value equal to the mean value within the admissible range, $h_{\mathrm{mean}}\;=\;\frac{h_{\min}+h_{\max}}{2}$.

The optimization strategies, to be described in the next subsection, are intended to find the best possible solution, not only for the altitude value, but also for the ground central point. It can be expected that, in the trivial case of a single target, both solutions will agree on the position of the vehicle located right on top of that target, at an altitude $h\;=\;h_{\min}$. In the case of two targets, both strategies should also come to the same solution, placing the vehicle on the central point of the segment that joins both targets, at a height $h\;=\;h_{\mathrm{mean}}$ (provided that, in the optimization algorithm, the same weighting is given to both terms and both cameras have equal focal length). Understandably, it is in more general cases—with an arbitrary number of targets and tracking cameras, where the best solution is not so predictable—that an optimization strategy is of real interest.

### 4.2. Setting up a Suitable Optimization Index

The key idea is that the proposed optimization index shall comprise a term for each target-tracking camera pair. In turn, each of these terms will include two quadratic subterms, one that weights the distance to the particular target and another that considers the verticality of the viewpoint to that same target:

$$J:=\sum_{i=1}^{n}\frac{1}{2}\;\left(J_{d_{i}}+J_{\gamma_{i}}\right)$$

Actuators will allow the vehicle to move and, consequently, to intently alter the value of the index, according to its gradient, until the optimum value is reached. Naturally, this is a dynamic process, since the targets can be subject to permanent motion.

Regarding the term related to verticality, $J_{\gamma_{i}}$, this is parameterized as a function of the angle $\gamma_{t_{i}}$, defined in (6). Specifically, we wish to minimize: $\tan^{2}\gamma_{t_{i}}$. This function takes value in the range [0…∞); its optimal value, 0, corresponds to a perfectly zenithal point of view, while, for a completely frontal point of view of the target, *∞* would be reached. If we move the vehicle over a horizontal plane with respect to a static target below it, progressively increasing the distance between the target and the ground-plane point above which the camera is located (in Figure 7, this distance was called $l_{i}$ and the referred point was represented in orange), we see that, as the minimum altitude is restricted to a value $h_{\min}>0$, the limiting value *∞* is only reached asymptotically, for a distance $l_{i}\to \infty$. We will be interested in weighting this term of the minimization index, by means of a coefficient $\epsilon_{\gamma }$, so that the value 1 corresponds to a limit of verticality bordering on the acceptable but yet attainable. To give some quantitative idea, let us suppose that this reference verticality limit is set to: $\gamma_{\lim}\;=\;70\frac{\pi }{180}\;$[rad]. If a constant height $h=h_{\min}$ is kept, this verticality limit would be met when $l_{i}\approx 2.75\;h_{\min}$.

According to the previous considerations, we would write the expression corresponding to this term in the form:

(12) $$J_{\gamma_{i}}\;=\;\epsilon_{\gamma }\;\tan^{2}\gamma_{t_{i}}\;;\;\epsilon_{\gamma }=\frac{1}{\tan^{2}\gamma_{\lim}}$$

Regarding the first term of the index, $J_{d_{i}}$, it could be thought as a function of the distance to the target, $d_{i}$:

(13) $$d_{i}\;:=\;∥{}^{c_{i}}p_{t_{i}}∥\;\approx \;∥{}^{b}p_{t_{i}}{∥=∥}^{w}\left({}^{b}p_{t_{i}}\right)∥$$

This approximation does not imply any loss of generality, since, knowing the orientation of the vehicle and the relative position of the nodal point of each camera with respect to the vehicle’s body, the exact expression could be used.

Since we are truly interested in monitoring or surveillance, we can assume that the absolute distances play a secondary role, though, while the observation quality or resolution is much more relevant. This being the case, a dimensionless measure of distance that conditions the observation quality might be helpful. In particular, we can define, for each tracking camera, the *optical range* as the ratio between the true distance to the target and the current focal length:

$$d_{\;f_{i}}\;:=\;\frac{d_{i}}{f_{i}}$$

with $f_{i}$ being the focal length of the *i*-th tracking camera. This gives greater flexibility to the feasible optimal solutions, as the index can automatically trade larger Euclidean distances for longer focal lengths.

Term $J_{d_{i}}$ will be defined in such a way that it introduces the optical range with a proper balance with respect to the verticality term already defined, as described next.

#### 4.2.1. Balancing Both Terms of the Optimization Index

The verticality term, $J_{\gamma_{i}}$, was defined in such a way that it reaches its minimum value, 0, for optimal verticality and a normalized value, 1, for the worst “admissible” verticality level, specified by $\gamma_{\lim}$. In a similar way, the optical range term, $J_{d_{i}}$, reaches its minimum value, 0, for optimal optical range, corresponding to the minimum distance, $h_{\min}$, while $\epsilon_{d}$ has to be defined such that this term reaches the normalized unitary value for the limit “admissible” optical range to be specified.

Let us consider the *i*-th target of interest of arbitrary shape, with a plant area in the real world $A_{i}\;\left[m^{2}\right]$. An approximate measure, independent of its particular shape, can be defined in the form of an equivalent diameter as: $d_{i}=2\sqrt{A_{i}\;/\;\pi }$. We can define the *projection ratio* as the quotient:

$$r_{i}=\frac{d_{i}^{im}}{d_{i}}$$

where $d_{i}^{im}$ is the equivalent diameter of the target projected on the image plane (also in [m]). This ratio is, of course, dependent of the distance to the target and the used focal length. In fact, it is the inverse of the previously introduced optical range:

$$r_{i}=\frac{1}{d_{\;f_{i}}}$$

This is a convenient parameter whose limits can be a priori fixed, with independence of the particular parameters of the camera to be used to track that target. This way, for our intended normalization of term $J_{d_{i}}$, desired limit values of projection ratio will be defined: $r_{\;\min}$, $r_{\;\max}$. After them, the term $J_{d_{i}}$ can be defined as:

$$J_{d_{i}}\;=\;\frac{{\left(d_{\;f_{i}}-1\;/\;r_{\;\max}\right)}^{2}}{{\left(1\;/\;r_{\;\min}-1\;/\;r_{\;\max}\right)}^{2}}$$

That can be rewritten as:

$$J_{d_{i}}\;=\;\frac{r_{\;\max}^{2}\;r_{\;\min}^{2}}{{\left(r_{\;\max}\;-r_{\;\min}\right)}^{2}}\;{\left(d_{\;f_{i}}-1\;/\;r_{\;\max}\right)}^{2}$$

A particularization of this can guarantee that, for all tracking cameras, the maximum projection ratio is reachable, and hence $J_{d_{i}}\;=\;0$ could be attained. Since the minimum allowed distance is $h_{\min}$, instead of specifying any given a priori value for $r_{\;\max}$, it can be defined after this minimum distance:

(14) $$r_{\;\max}=\frac{f_{i}}{h_{\min}}$$

From this, a particular expression of $J_{d_{i}}$ can be derived:

(15) $$J_{d_{i}}\;=\;\epsilon_{d}\;\frac{{\left(d_{i}-h_{\min}\right)}^{2}}{{\left(f_{i}-r_{\;\lim}\;h_{\min}\right)}^{2}}\;;\;\epsilon_{d}=r_{\;\lim}^{2}$$

Here, for parallelism with definition (12), $r_{\;\lim}$, equivalent to $r_{\;\min}$, has been introduced, making reference to the ”admissible” minimum value of the projection ratio.

**Figure 8:**
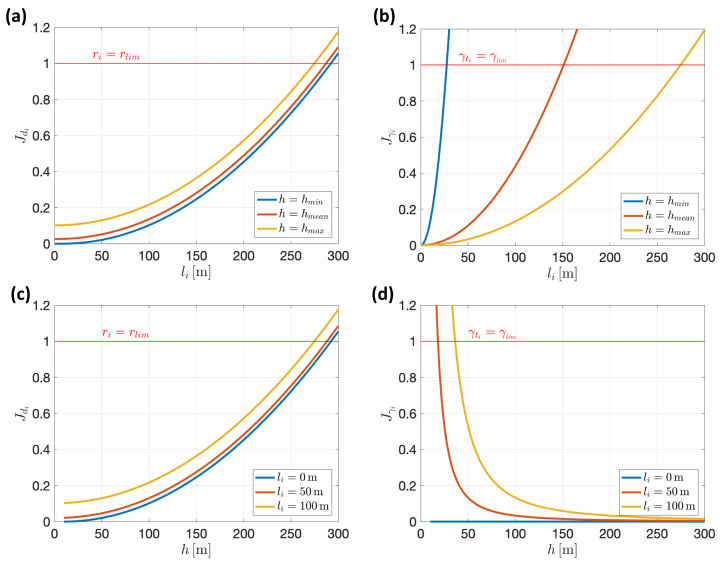
Achieved trade-off between both optimization terms. (**a**,**b**) Comparison of terms $J_{d_{i}}$ and $J_{\gamma_{t_{i}}}$, respectively, for varying projected distance $l_{i}$, under three different values of constant altitude. (**c**,**d**) Comparisons of the same terms for varying altitudes, under three different values of constant $l_{i}$ distance.

Figure 8 allows to assess the achieved trade-off between both terms, given by expressions (12) and (15). The top plots show their evolution as a function of $l_{i}$ under constant altitude, $h\;=\;\{h_{\min},\;h_{\mathrm{mean}},\;h_{\max}\}$. In this analysis, $h_{\min}\;\;=\;10$ [m], $h_{\max}\;\;=\;100$ [m], $\gamma_{\lim}\;=\;70\frac{\pi }{180}\;$[rad], $r_{\;\lim}\;\;=\;\frac{r_{\;\max}}{30}$ and $f_{i}\;\;=\;50\;\times \;10^{-3}$ [m]. The bottom plots show a similar comparison as a function of *h* under constant $l_{i}$ distance: $l_{i}\;=\;\left\{0,\;50,\;100\right\}$ [m].

#### 4.2.2. Uniform Optimization Index

As a conclusion of the above, the following index is proposed as the one to be minimized:

(16) J(di,γti):=∑i=1nϵd2di−hmin2fi−rlimhmin2+ϵγ2tan2γti⏟Ji(di,γti)s.t.hmin≤h≤hmax

where the factor $\frac{1}{2}$ has been introduced for convenience and where $\epsilon_{d}$ and $\epsilon_{\gamma }$ are:

$$\epsilon_{d}=r_{\;\lim}^{2}\;;\;\epsilon_{\gamma }=\frac{1}{\tan^{2}\gamma_{\lim}}$$

In the event that, for some specific reason, it is desired to give more prevalence to some targets, different values could be given to the weighting coefficients of the different terms. For this, it would suffice to replace, in the previous expression, the coefficients $\epsilon_{d},\;\epsilon_{\gamma }$ by the corresponding $\epsilon_{d_{i}}\;=\;\epsilon_{i}\;\epsilon_{d},\;\epsilon_{\gamma_{i}}\;=\;\epsilon_{i}\;\epsilon_{\gamma }$, where $\epsilon_{i}$ gives the particular relative weighting factor for the *i*-th target, satisfying $\sum_{i=1}^{n}\epsilon_{i}=1$.

**Figure 9:**
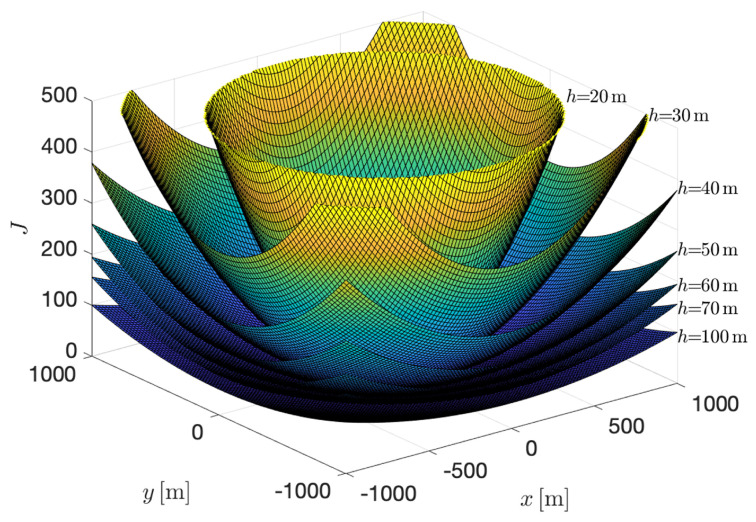
Shape of the hypersurface *J* for a discrete set of altitudes.

**Figure 10:**
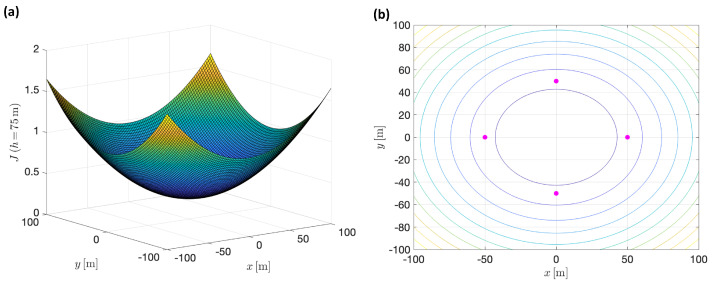
(**a**) Particularizing the hypersurface *J* for altitude h=75 [m]. (**b**) Corresponding level curves. Magenta dots represent targets’ locations.

In order to give an example of the behavior of this index, a scenario with a set of four targets, located in symmetric positions with respect to a central point in the ground plane, using equal focal length for each tracking camera, is explored in Figure 9. This figure shows the appearance of the hypersurface *J* as a function of the vehicle’s position in space, relative to the location of the targets (origin of plane X−Y is the central point of the targets’ positions). For a particular altitude, h=70, the corresponding surface and level curves are shown in Figure 10.

In this experiment, the allowed altitude interval was defined from $h_{\min}\;=\;10$ [m] to $h_{\max}\;=\;100$ [m]; the targets were stationary, at positions ${}^{w\;}p_{t_{1}}\;\;=\;\;{\left[50,0,0\right]}^{\;⊤}$ y ${}^{w\;}p_{t_{2}}\;\;=\;\;{\left[-50,0,0\right]}^{\;⊤}$, ${}^{w\;}p_{t_{3}}\;\;=\;\;{\left[0,50,0\right]}^{\;⊤}$ y ${}^{w\;}p_{t_{4}}\;\;=\;\;{\left[0,-50,0\right]}^{\;⊤}$. The focal length of each one of the tracking cameras was set equal to $f_{i}\;=\;50\;$[mm]. The specific altitude at which the optimum value of the index is reached is h=74.8 [m]. It can be verified that, as expected, this same solution is obtained for an arbitrary number of targets, symmetrically distributed around a central point (graphically normalized as the origin).

The proposed optimization index (16) ultimately depends on the relative position between the vehicle and each particular target:

(17) $$J_{i}\left({}^{b}p_{t_{i}}\right)$$

Considering that the verticality is referred to inertial axes and that the relative target-vehicle distance (13) depends, in turn, on the respective absolute positions:

$${}^{w\;}\left({}^{b}p_{t_{i}}\right)={}^{w\;}p_{t_{i}}-{}^{w\;}p_{b}$$

Once the positions of the targets have been estimated through the geolocation process described in Section 3.4, the index will ultimately have the position ${}^{w\;}p_{b}$ as the only variable:

$$J\;=\;J({}^{w}\;p_{b})$$

The optimization problem, therefore, boils down to computing the vehicle’s position that provides the minimum value of the index, subject to the altitude interval:

(18) wpb*=argminwpb∑i=1nϵd2di−hmin2fi−rlimhmin2+ϵγ2tan2γtis.t.hmin≤h≤hmax

Although it does not seem to offer a straightforward analytical solution, it can be proven that the function to be minimized is convex (see Appendix A), so that a single global minimum exists. Therefore, standard numerical methods to find the minima of constrained nonlinear multivariable functions will be used.

In view of the convex nature of the hypersurface at hand, an arbitrary starting point for the iterative algorithm could be taken. However, in order to help speed up the numerical computation, it would be interesting to have some criteria for the choice of the initial condition. For this reason, a variant of the index is analyzed in the next subsection, providing an approximation that does have a closed-form solution.

#### 4.2.3. Approximate Uniform Optimization Index

For this approximation of the uniform index, we simply redefine $J_{d_{i}}$ in (15) as follows:

(19) $$J_{d_{i}}^{\prime }\;=\;\epsilon_{d}\;\frac{\left(d_{i}^{2}-h_{\min}^{2}\right)}{{\left(f_{i}-r_{\lim}\;h_{\min}\right)}^{2}}$$

$$J_{i}^{\prime }=J_{d_{i}}^{\prime }+J_{\gamma_{i}}$$

After this, expression (18) can be rewritten as:

(20) wpb*′=argminwpb∑i=1nϵd2di2−hmin2fi−rlimhmin2+ϵγ2tan2γtis.t.hmin≤h≤hmax

The index, in this case, gives rise to a similar convex hypersurface, which can be taken as an approximation to the original one. In fact, it can be readily seen that the difference between $J_{i}^{\prime }$ given by (19) and $J_{i}$ in (15) is strictly proportional to $\frac{d_{i}}{h_{\min}}$, but with the particularity that its relative value $\frac{|J_{d_{i}}^{\prime }-J_{d_{i}}|}{J_{d_{i}}}$ rapidly converges to zero as the ratio $\frac{d_{i}}{h_{\min}}$ increases.

An analytic solution is available for this approximate index (the detailed development is given in Appendix B). The conclusion of that study is that the first two components are specific weighted averages of the respective coordinates of the target locations, while the altitude is a function that depends on the scattering of the targets on the ground plane. The attained expressions for the components of ${}^{w}\;p_{b}^{{*}^{\prime }}={\left[{}^{w}\;x_{b}^{{*}^{\prime }},\;{}^{w}\;y_{b}^{{*}^{\prime }},\;{}^{w}\;z_{b}^{{*}^{\prime }}\right]}^{\;⊤}$ are as follows:

$${}^{w}\;x_{b}^{{*}^{\prime }}=\frac{\sum_{i=1}^{n}\;\left(\frac{\epsilon_{d}}{{\left(f_{i}-r_{\lim}\;h_{\min}\right)}^{2}}\;+\;\frac{\epsilon_{\gamma }}{h^{2}}\right)\;{}^{w}\;x_{t_{i}}}{\sum_{i=1}^{n}\;\frac{\epsilon_{d}}{{\left(f_{i}-r_{\lim}\;h_{\min}\right)}^{2}}\;+\;\frac{\epsilon_{\gamma }}{h^{2}}\;n}$$

$${}^{w}\;y_{b}^{{*}^{\prime }}=\frac{\sum_{i=1}^{n}\;\left(\frac{\epsilon_{d}}{{\left(f_{i}-r_{\lim}\;h_{\min}\right)}^{2}}\;+\;\frac{\epsilon_{\gamma }}{h^{2}}\right)\;{}^{w}\;y_{t_{i}}}{\sum_{i=1}^{n}\;\frac{\epsilon_{d}}{{\left(f_{i}-r_{\lim}\;h_{\min}\right)}^{2}}\;+\;\frac{\epsilon_{\gamma }}{h^{2}}\;n}$$

$${}^{w}\;z_{b}^{{*}^{\prime }}=h={\left[\frac{\epsilon_{\gamma }\;n}{\epsilon_{d}\;\sum_{i=1}^{n}\frac{1}{{\left(f_{i}-r_{\lim}\;h_{\min}\right)}^{2}}}\;\mathrm{tr}\left(V^{•}\right)\right]}^{\frac{1}{4}}$$

Regarding the first two components of the solution, the weighting coefficients, however, are not constant, since they depend on the resulting value for the altitude itself. If we give the name $\mu^{•}$ to this particular average of the target locations on the ground plane, $\mu^{•}:={\left[{}^{w}\;x_{b}^{{*}^{\prime }},\;{}^{w}\;y_{b}^{{*}^{\prime }},\;0\right]}^{\;⊤}$, $V^{•}$, defining the third component, is the covariance matrix of the set of targets’ positions with respect to the defined average $\mu^{•}$:

$$V^{•}=\frac{1}{n}\;\sum_{i=1}^{n}\left({}^{w}\;p_{t_{i}}\;-\;\mu^{•}\right){\left({}^{w}\;p_{t_{i}}\;-\;\mu^{•}\right)}^{\;⊤}$$

The fact that the optimal altitude depends on the covariance matrix also has an easy intuitive interpretation. The more scattered the set of targets is, the higher the altitude to be adopted, in order to achieve a better overall verticality level. This solution provides a tangible expression for this intuitive idea.

It is interesting to see that, in the particular case of all the tracking cameras having the same focal lengths, $f_{i}\;=\;f$, the complete analytical expression for the optimal position of the vehicle, according to the approximate index, is reduced to:

$${}^{w}\;p_{b}^{{*}^{\prime }}\;=\;\left[\begin{array}{c} e_{1}^{\;⊤}\;\mu \\ e_{2}^{\;⊤}\;\mu \\ {\left[\frac{\epsilon_{\gamma }}{\epsilon_{d}}\;{\left(\frac{f}{r_{\lim}}\;-\;\;h_{\min}\right)}^{2}\;\mathrm{tr}\left(V\right)\right]}^{\frac{1}{4}} \end{array}\right]\;\;if\;f_{i}\;\;=\;\;f\;,\;\forall \;i\;=\;1,\ldots ,n$$

where μ and V are the conventional mean and covariance matrix of the target locations, respectively:

$$\mu =\frac{1}{n}\;\sum_{i=1}^{n}{}^{w}\;p_{t_{i}}$$

$$V=\frac{1}{n}\;\sum_{i=1}^{n}\left({}^{w}\;p_{t_{i}}\;-\;\mu \right){\left({}^{w}\;p_{t_{i}}\;-\;\mu \right)}^{\;⊤}$$

That is, as far as the first two components are concerned, the optimal solution takes the average of the target positions on the ground plane, as expected. Moreover, this is true regardless of the specific weightings adopted for the distance and verticality optimal terms, $\epsilon_{d}$ and $\epsilon_{\gamma }$. This is so because, in that particular case, the introduced optical range is equivalent to the Euclidean distance. Reflecting briefly on this, although the a priori choice of the center of mass for these two coordinates might have seemed, although logical, somewhat arbitrary, there is now a mathematical foundation to support it, under conditions of equal focal lengths. For the altitude, however, we did not have any a priori choice of the sort. This approximate index also provides an analytical expression for it, with an interpretation according to intuitive reasoning.

Going back to our motivation for the introduction of the approximate index, this analytical solution would be taken as the initial value for the iterative algorithm that searches for the optimum of the original uniform index (18). This will be the case as long as the preliminary analytical solution satisfies the constraints. Accordingly, we can establish the following initial condition for the original uniform optimization index:

$${}^{w}\;p_{b}^{*}\left(0\right)=\left[\begin{array}{c} {}^{w}\;x_{b}^{{*}^{\prime }}\\ {}^{w}\;y_{b}^{{*}^{\prime }}\\ \max\left(\min\left({}^{w}\;z_{b}^{{*}^{\prime }},\;h_{\max}\right),\;h_{\min}\right) \end{array}\right]$$

#### 4.2.4. Min–Max Optimization Index

The first form introduced for the optimization index, given in (18), is referred as *uniform optimization*, in the sense that no special relevance is given to any of the targets. This is one of the reasons why some sort of weighted means of the target coordinates came out “in the way” to the solution, at least concerning its attained approximated analytical form. We can, however, reframe our idea of the optimal solution, attempting to guarantee, not the best average point of view, but to optimize the worst point of view. With this, we come back to our uniform versus periphery-biased discussion in Section 4.1, where the outermost targets were given a special relevance. In order to provide a formal framework in this case, the proposal could be formulated as a min–max optimization problem:

(21) wpb*=argminwpbmaxiϵd2di−hmin2fi−rlimhmin2+ϵγ2tan2γtis.t.hmin≤h≤hmax

In this case, we are changing the index given in the expression (16) by the following:

(22) $$J\;:=\;\max_{i}\left(\frac{1}{2}\;\epsilon_{d}\;\frac{{\left(d_{i}-h_{\min}\right)}^{2}}{{\left(f_{i}-r_{\lim}\;h_{\min}\right)}^{2}}\;+\;\frac{1}{2}\;\epsilon_{\gamma }\;\;\tan^{2}\gamma_{t_{i}}\right)$$

**Figure 11:**
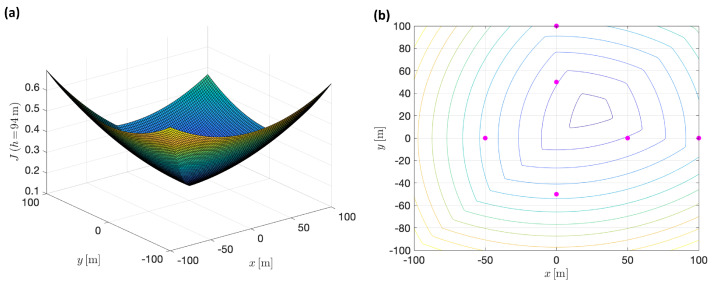
(**a**) Min–max surface example, corresponding to a particular altitude (h=94 [m]), for a given non-symmetric six-target arrangement. (**b**) Corresponding level curves, where the magenta dots represent targets’ locations.

As expected, under general target configurations, the hypersurface is different and the results are different. An example of the surface appearance and the corresponding level curves for a given value of altitude is shown in Figure 11, in contrast to Figure 10. At first sight, perhaps the most obvious difference is that the level curves are not elliptical in this case. This particular example corresponds to the same conditions of Figure 10, but with two additional targets, located at positions: ${}^{w\;}p_{t_{5}}\;=\;{\left[100,0,0\right]}^{\;⊤}$, ${}^{w\;}p_{t_{6}}\;=\;{\left[0,100,0\right]}^{\;⊤}$. Again, all the tracking cameras share the same focal length, $f_{i}\;=\;50\;$[mm].

This is also a convex hypersurface (see Appendix A), again with no feasible closed-form solution.

In the absence of an analytically-tractable approximation with a closed-form solution, as in the uniform case, a convenient initial condition can be obtained, at least partially, following some ideas of the corresponding baseline strategy. For instance, the first two components of ${}^{w}\;p_{b}^{*}\left(0\right)$ can be taken as the center of the convex hull of the target configuration. Regarding the altitude, the center of the acceptable altitude interval can be used as the initial condition, or the analytic solution provided by the approximate uniform index.

## 5. Results and Discussion

This section presents some simulations to comparatively assess the performance of the different positioning strategies described in the work, from the baseline alternatives to those raised by optimization indices.

Our simulated scenarios are built under MATLAB, consisting in one agent mounting one central camera and four tracking cameras (n=4). The central camera is located right under the geometric center of the vehicle’s frame, being modeled as a wide-angle camera with 2.8 mm focal length. The tracking cameras are symmetrically distributed around the central one, along the X and Y vehicle’s frame axes. These cameras feature much longer focal lengths, in particular 50 mm or 20 mm will be used. The transitory response in the own agent’s displacement or the gimbal steering control is considered negligible, so their effects will not be apparent in the simulations.

In the two experiments described in this section, the reference positions supplied by the different strategies at each instant are comparatively evaluated. The two proposed optimal approaches are denoted as *OptUniform* and *OptMinMax*, referring to the uniform optimal approach, given by expression (18), and the min–max optimal approach, given ty expression (21), respectively. Common optimization functions available in MATLAB, such as *fmincon()* and *fminimax()*, are used to implement these strategies, including the altitude constraints. In the case of *fminimax()*, the tolerance has been reduced to $10^{-12}$ for better results. Additionally, both baseline strategies, uniform and periphery-biased, described in Section 4.1, are also included. Please recall that the first one, referred to as *NaiveUniform*, simply takes, as the desired instantaneous vehicle’s position, the center of mass of the estimated positions of the targets on the ground plane at a constant altitude, to be specified. The second one, referred to as *NaiveConvexHull*, selects instead the center of the convex hull defined by the set of targets, again at a constant altitude. In both cases, the chosen predefined altitude is $h_{\mathrm{mean}}$.

**Figure 12:**
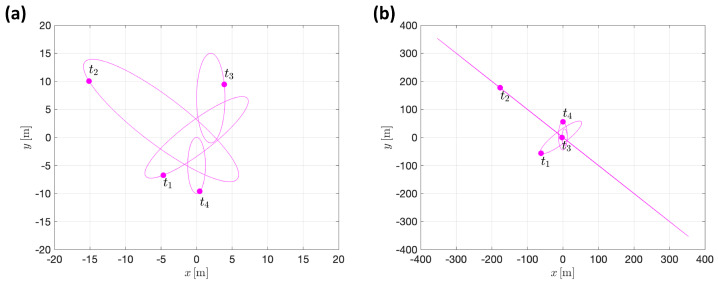
(**a**) Simulated target trajectories for Experiment 1. (**b**) Corresponding target trajectories for Experiment 2.

In the fist experiment, $f_{i}\;=\;50\;\mathrm{mm}\;\forall \;i\;=\;\left\{1,3,4\right\}$, while $f_{2}\;=\;20$ mm, $h_{\min}\;\;=\;10$ [m], $h_{\max}\;=\;100$ [m], $\gamma_{\lim}\;=\;70\;\frac{\pi }{180}\;$[rad] and $r_{\lim}\;\;=\;1.66\times 10^{-4}$. The corresponding four targets move along closed elliptic trajectories, with different centers, amplitudes, and velocities. The particular trajectories followed by the targets during this first experiment are shown in the left plot of Figure 12, being their respective velocities: $v_{t_{1}}\;\;=\;v_{t_{2}}\;\approx \;2.1\;\left[\frac{m}{s}\right]$, $v_{t_{3}}\;\approx \;0.86\;\left[\frac{m}{s}\right]$ and $v_{t_{4}}\;\approx \;0.43\;\left[\frac{m}{s}\right]$.

**Figure 13:**
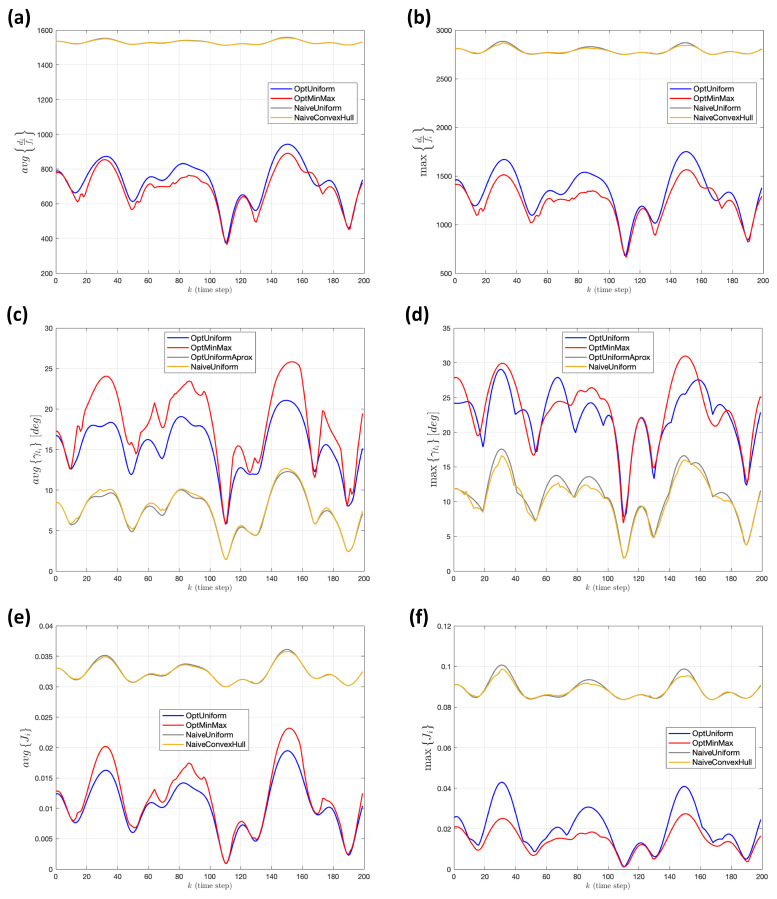
Experiment 1. (**a**,**b**) Instantaneous mean and maximum values in *optical range*, respectively. (**c**,**d**) Corresponding mean a maximum measures for the *deficit in verticality*. (**e**,**f**) Mean and maximum values of the set of individual optimization terms $J_{i}$.

In order to assess the suitability of the different strategies, Figure 13 provides a comparison, during 200 time steps ($T_{m}=0.5\;$ s), in the instantaneous values of the respective *optical range* ($\frac{d_{i}}{f_{i}}$) and *deficit of verticality* ($\gamma_{t_{i}}$), in terms of their mean and maximum values. We can see that both naive strategies are very close to each other. This is due to the fact that, with only four targets, most of the time the convex hull will indeed correspond to the polygon defined by all four targets, making its center equivalent to the center of mass. On the other hand, both naive strategies provide very unbalanced behavior among optical range and verticality. In this particular experiment, as it turns out that the predefined altitude $h_{\mathrm{mean}}$ is too high, according to the range of displacement of the targets, verticality strongly prevails, at the expense of distance. The bottom plots of the figure help in gauging how far from the respective optimal trade-off the different approaches lead the vehicle, when these two factors are simultaneously taken into account, as in each term of the optimization index $J_{i}$ in (16).

**Figure 14:**
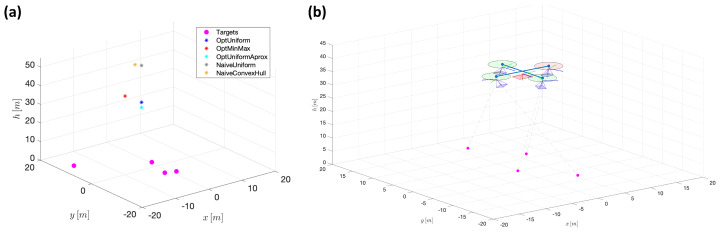
(**a**) Example of reference position provided for each positioning strategy. (**b)** Snapshot of the three-dimensional scenario depicting the vehicle.

A graphic animation of Figure 14, showing the instantaneous reference position for each one of the strategies is included as part of the Supplementary Material (the corresponding filename is *exp1_videoRefPositions3DSubplots.avi*). The right plot of the figure shows a snapshot of the described scenario, depicting a quadcopter-type agent (central camera in red, tracking cameras in blue), flying over the set of targets. Again, as part of the Supplementary Material, a video demonstrating the simulated behavior of the agent is included (the filename is *exp1_videoChameleon3DSubplots.avi*, where the agent follows the position reference provided by the *OptUniform* strategy).

**Figure 15:**
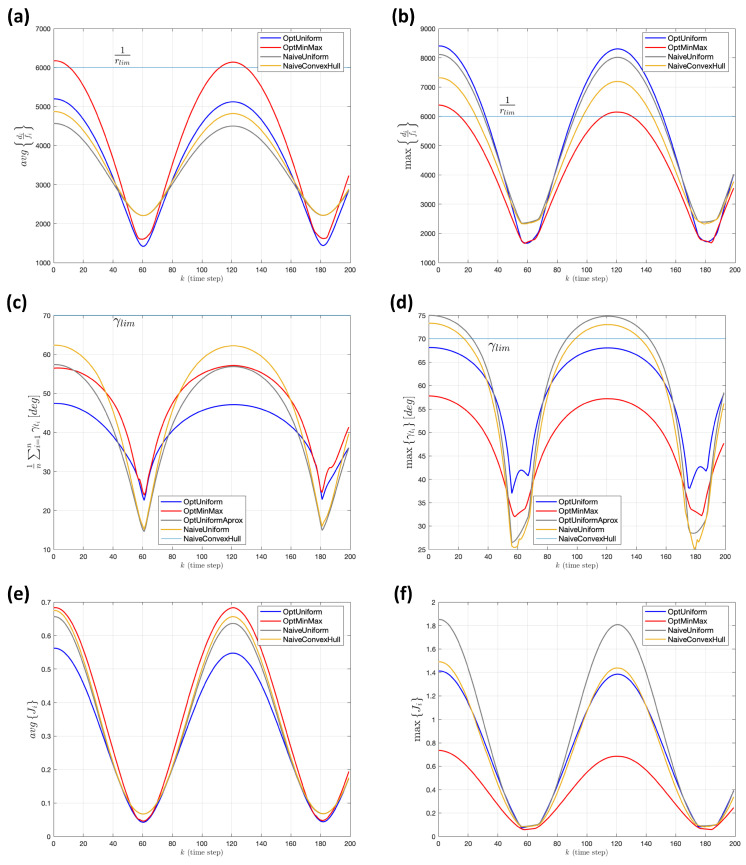
Experiment 2. (**a**,**b**) Instantaneous mean and maximum values in *optical range*, respectively. (**c**,**d**) Corresponding mean a maximum measures for the *deficit in verticality*. (**e**,**f**) Mean and maximum values of the set of individual optimization terms $J_{i}$.

For the second experiment, a more challenging situation is simulated, where the second target moves back and forth over a straight line, reaching points very far from the other three targets, as shown in the right plot of Figure 12. The target velocities in this second scenario are: $v_{t_{1}}\;\approx \;4.3\;\left[\frac{m}{s}\right]$, $v_{t_{2}}\;\approx \;16.6\;\left[\frac{m}{s}\right]$, $v_{t_{3}}\;\approx \;0.8\;\left[\frac{m}{s}\right]$ and $v_{t_{4}}\approx \;1.05\;\left[\frac{m}{s}\right]$. In this case, all of the tracking cameras share the same focal length, $f_{i}\;=\;50\;\mathrm{mm}\;\forall \;i\;=\;\left\{1,2,3,4\right\}$. The altitude limits are set to the following values: $h_{\min}\;\;=\;10$ [m], $h_{\max}\;\;=\;200$ [m], while $r_{\lim}$ and $\gamma_{\lim}$ remain unchanged. Figure 15 shows the comparison in the instantaneous mean and maximum values of optical range and verticality, together with the combined measure, based on the set of $J_{i}$ values. In this case, some of the strategies reach the so called “admissible” limit values for the projection ratio, $r_{\lim}$, or verticality, $\gamma_{\lim}$. However, it is clear that the reference positions provided by both optimal approaches show the best behavior according to their respective strategy, mean-based versus maximum-based minimization. Again, as part of the Supplementary Material, a video file is provided, demonstrating this experiment (the filename is *exp2_videoRefPositions3DSubplots.avi*, where the agent follows the position reference provided by the *OptUniform* strategy).

From a practical point of view, there is another differential aspect between both optimization algorithms that can be noticed. Namely, the fact that the min–max optimization algorithm might exhibit frequent abrupt changes in the velocity of the reference position provided as a solution, even when the targets move along smooths trajectories, as in the previous experiments. This is an expected behavior, derived from the sudden changes in the identity of the most outlying target at each instant. In any case, it will be the responsibility of the own agent’s trajectory planner to avoid any inconvenience arising from this fact.

## 6. Conclusions

In the context of autonomous surveillance and multi-target tracking applications, this paper discusses some of the possibilities offered by the presence of multiple independently steerable cameras onboard unmanned aerial agents, as a means to provide higher levels of efficiency with minimal resource deployment. Some of the implications are thoroughly described, including known aspects, e.g., target geolocation, target aiming, and target allocation, which are presented under the new perspective. The problem of the optimal positioning of a single aerial agent with respect to a set of moving targets, operating under the proposed paradigm, is specifically addressed, suggesting suitable performance functions.

Instead of restricting the agent’s motion to a plane of constant altitude, the altitude itself is formulated as part of the solution, by means of introducing verticality of the viewpoints along with the distance to the targets, as desirable qualities to be balanced in an optimal way. Moreover, since the focal lengths of the multiple onboard cameras may, in general, differ from each other, the notion of distance needs to be adapted into a notion of *optical range*, as the agent can trade longer Euclidean distances for correspondingly longer focal lengths.

The work can be seen as a first theoretical approach to the problems raised, including simulation studies aiming to validate its feasibility, under the action of the suggested optimization indices. Future lines of research will focus on target clustering, as an immediate extension of the one-to-one scenario here addressed. Moreover, the introduction of the camera zooming factors into the optimization problem itself can be seen as a most compelling immediate future line of research.

## Supplementary Materials

The following supporting information can be downloaded at: https://www.mdpi.com/article/10.3390/s22062359/s1.

## Appendix A. Uniform and Min–Max Optimization Indices: Convexity Proofs

The convexity proof of the uniform optimization index is provided in the form of the following theorem:

**Theorem** **A1.**
*The optimization index is defined as*

(A1)
$$J=\sum_{i=1}^{i=n}\;\left[\frac{\epsilon_{d}}{2}\frac{{\left(d_{i}-h_{\min}\right)}^{2}}{{\left(f_{i}-r_{\lim}\;h_{\min}\right)}^{2}}+\frac{\epsilon_{\gamma }}{2}\tan^{2}\gamma_{t_{i}}\right]$$

*is the convex on the position of the UAV’s main body, ${}^{w\;}p_{b}\;\in \;S$ with $S=\{p\;\in \;R^{3}\;/\;e_{3}^{\;⊤}\;p>e_{3}^{\;⊤}\;{}^{w\;}p_{t_{i}}$∀i=1,…,n}.*

**Proof.** Equation (A1) can be explicitly expressed in terms of the position of the vehicle’s main body ${}^{w\;}p_{b}$ and the targets ${}^{w\;}p_{t_{i}}$ as

(A2) $$J=\sum_{i=1}^{i=n}\;\left[\frac{\epsilon_{d}}{2}\;J_{d_{i}}^{u}+\frac{\epsilon_{\gamma }}{2}\;J_{\gamma_{i}}^{u}\right]$$

with $J_{d_{i}}^{u}$ and $J_{\gamma_{i}}^{u}$ being the unweighted core quadratic functions, which can be written as:

(A3) $$\begin{array}{ccc} J_{d_{i}}^{u} & = & \frac{{\left({∥}^{w\;}p_{b}\;-\;{}^{w}\;p_{t_{i}}∥-h_{\min}\right)}^{2}}{{\left(f_{i}-r_{\lim}\;h_{\min}\right)}^{2}} \end{array}$$

(A4) $$\begin{array}{ccc} J_{\gamma_{i}}^{u} & = & \frac{∥P_{\;12}\;{(}^{w\;}p_{b}-{}^{w}\;p_{t_{i}}){∥}^{2}}{∥P_{\;3}\;\left({}^{w}\;p_{b}\;-\;{}^{w}\;p_{t_{i}}\right){∥}^{2}} \end{array}$$

and the projection matrices $P_{\;12}$ and $P_{\;3}$ defined as $P_{\;12}={\left[e_{1}\;\;e_{2}\;\;0\right]}^{\;⊤}$ and $P_{\;3}={\left[0\;\;0\;\;e_{3}\right]}^{\;⊤}$ To proof convexity of (A2), we will first verify that individual functions $J_{d_{i}}^{u}$ and $J_{\gamma_{i}}^{u}$ are convex for i=1,…,n. Convexity of $J_{d_{i}}^{u}$ is straightforward as it is well known that the two-norm is a convex function, and so it is a squared affine combination of the norm, as the definition of $J_{d_{i}}^{u}$ states. To “proof” the convexity of $J_{\gamma_{i}}^{u}$, let us express the components of ${}^{w\;}p_{b}\;\in \;R^{3}$ and ${}^{w\;}p_{t_{i}}\;\in \;R^{3}$ in the {W} frame as ${}^{w\;}p_{b}={{[}^{w\;}x_{b}\;{\;}^{w\;}y_{b}\;{\;}^{w\;}z_{b}]}^{\;⊤}$ and ${}^{w\;}p_{t_{i}}={{[}^{w\;}x_{t_{i}}\;{\;}^{w\;}y_{t_{i}}\;{\;}^{w\;}z_{t_{i}}]}^{\;⊤}$. Thus, we can rewrite

$$J_{\gamma_{i}}^{u}=\frac{{{(}^{w\;}x_{b}{-}^{w\;}x_{t_{i}})}^{2}+{{(}^{w\;}y_{b}{-}^{w\;}y_{t_{i}})}^{2}}{{{(}^{w\;}z_{b}{-}^{w\;}z_{t_{i}})}^{2}}$$

$J_{\gamma_{i}}^{u}$ is continuous twice-differentiable in S (notice that the definition of the set S accounts for the hypothesis that the UAV is assumed to be always positioned above all targets with respect to frame {W}, the condition that guarantees that the term ${}^{w\;}z_{b}{-}^{w\;}z_{t_{i}}$ is strictly positive for all targets *i*), so its convexity is verified if and only if the (symmetric) Hessian matrix $\nabla^{2}J_{\gamma_{i}}^{u}$ is positive semidefinite

$$\nabla^{2}J_{\gamma_{i}}^{u}=\left(\begin{array}{ccc} \frac{2}{{{(}^{w\;}z_{b}{-}^{w\;}z_{t_{i}})}^{2}} & 0 & -\frac{4{(}^{w\;}x_{b}{-}^{w\;}x_{t_{i}})}{{{(}^{w\;}z_{b}{-}^{w\;}z_{t_{i}})}^{3}}\\ {}* & \frac{2}{{{(}^{w\;}z_{b}{-}^{w\;}z_{t_{i}})}^{2}} & -\frac{4{(}^{w\;}y_{b}{-}^{w\;}y_{t_{i}})}{{{(}^{w\;}z_{b}{-}^{w\;}z_{t_{i}})}^{3}}\\ {}* & * & \frac{12({{(}^{w\;}x_{b}{-}^{w\;}x_{t_{i}})}^{2}+{{(}^{w\;}y_{b}{-}^{w\;}y_{t_{i}})}^{2})}{{{(}^{w\;}z_{b}{-}^{w\;}z_{t_{i}})}^{4}} \end{array}\right)$$

this condition is easily verified as

$$det\left(\nabla^{2}{J_{\gamma }}_{i}\right)=\frac{16\;({{(}^{w\;}x_{b}{-}^{w\;}x_{t_{i}})}^{2}+{{(}^{w\;}y_{b}{-}^{w\;}y_{t_{i}})}^{2})}{{{(}^{w\;}z_{b}{-}^{w\;}z_{t_{i}})}^{8}}\geq 0\;\mathrm{for}\;{{[}^{w\;}x_{b},{\;}^{w\;}y_{b},{\;}^{w\;}z_{b}]}^{\;⊤}\in S$$

The proof is completed from the definition of convex functions and the fact that (A2) is a weighted sum of 2n convex functions $J_{d_{i}}^{u}$ and $J_{\gamma_{i}}^{u}$ with positive coefficients $\frac{\epsilon_{d}}{2}$ and $\frac{\epsilon_{\gamma }}{2}$. Thus, for any $p\;\in \;R^{3}$, $q\;\in \;R^{3}$ and α∈R,0≤α≤1 we have

$$\begin{array}{ccc} J(\alpha \;p+(1\;\;-\;\;\alpha )\;q) & = & \sum_{i=1}^{i=n}\frac{\epsilon_{d}}{2}\;J_{d_{i}}^{u}\left(\alpha \;p+\left(1\;\;-\;\;\alpha \right)\;q\right)+\frac{\epsilon_{\gamma }}{2}J_{\gamma_{i}}^{u}\left(\alpha \;p+\left(1\;\;-\;\;\alpha \right)\;q\right)\\ & \leq & \sum_{i=1}^{i=n}\frac{\epsilon_{d}}{2}\left(\alpha \;J_{d_{i}}\left(p\right)+\left(1\;\;-\;\;\alpha \right)\;J_{d_{i}}\left(q\right)\right)+\frac{\epsilon_{\gamma }}{2}\left(\alpha \;J_{\gamma_{i}}\left(p\right)+\left(1\;\;-\;\;\alpha \right)\;J_{\gamma_{i}}\left(q\right)\right)\\ & = & \alpha \;J(p)+(1\;\;-\;\;\alpha )\;J(q) \end{array}$$

□

Regarding the min–max index given by expression (21), it can be trivially proved to be convex, as it is well known that the pointwise maximum of a collection of convex functions is convex. Both terms in the summation of (22) are convex, as proved by the previous theorem.

## Appendix B. Approximate Uniform Optimization Index: Gradient Derivation and Closed-Form Solution

Starting from the expression (20), which is reproduced here for convenience:

$${}^{w}\;p_{b}^{{*}^{\prime }}\;=\;\arg\min_{{}^{w}\;p_{b}}\;\sum_{i=1}^{n}\;\left[\frac{\epsilon_{d}}{2}\;\frac{\left(d_{i}^{2}-h_{\min}^{2}\right)}{{\left(f_{i}-r_{\lim}\;h_{\min}\right)}^{2}}\;+\;\frac{\epsilon_{\gamma }}{2}\;\tan^{2}\gamma_{t_{i}}\right]$$

where, for the analytical study, the constraints have been excluded. Considering the gradient of each of the two subterms involved in the *i*-th term of the index:

$$\nabla J_{i}^{\prime }\;=\;\frac{\partial J_{i}^{\prime }}{\partial d_{i}^{2}}\;\frac{\partial d_{i}^{2}}{\partial {}^{w}\;p_{b}}\;+\;\frac{\partial J_{i}^{\prime }}{\partial \tan^{2}\gamma_{t_{i}}}\;\frac{\partial \tan^{2}\gamma_{t_{i}}}{\partial {}^{w}\;p_{b}}$$

$$\frac{\partial J_{i}^{\prime }}{\partial d_{i}^{2}}=\frac{\epsilon_{d}}{2\;{\left(f_{i}-r_{\lim}\;h_{\min}\right)}^{2}}$$

$$\frac{\partial d_{i}^{2}}{\partial {}^{w}\;p_{b}}=-2\;\left({}^{w}\;p_{t_{i}}\;-\;{}^{w}\;p_{b}\right)$$

$$\frac{\partial J_{i}^{\prime }}{\partial \tan^{2}\gamma_{t_{i}}}=\frac{\epsilon_{\gamma }}{2}$$

$$\frac{\partial \tan^{2}\gamma_{t_{i}}}{\partial {}^{w}\;p_{b}}=-\frac{2}{h^{2}}\;\left[\begin{array}{c} \left({}^{w}\;x_{t_{i}}\;-\;{}^{w}\;x_{b}\right)\\ \left({}^{w}\;y_{t_{i}}\;-\;{}^{w}\;y_{b}\right)\\ \frac{{\left({}^{w}\;x_{t_{i}}\;-\;{}^{w}\;x_{b}\right)}^{2}+{\left({}^{w}\;y_{t_{i}}\;-\;{}^{w}\;y_{b}\right)}^{2}}{h} \end{array}\right]$$

In the second partial derivative, we made use of the fact that, for any vector v:

$$\frac{{\partial ∥v∥}^{2}}{\partial v}=2\;v$$

As a result, the *i*-th gradient vector adopts the form:

$$\nabla J_{i}^{\prime }=-\frac{\epsilon_{d}}{{\left(f_{i}-r_{\lim}\;h_{\min}\right)}^{2}}\;\left({}^{w}\;p_{t_{i}}\;-\;{}^{w}\;p_{b}\right)\;-\;\frac{\epsilon_{\gamma }}{h^{2}}\;\left[\begin{array}{c} \left({}^{w}\;x_{t_{i}}\;-\;{}^{w}\;x_{b}\right)\\ \left({}^{w}\;y_{t_{i}}\;-\;{}^{w}\;y_{b}\right)\\ \frac{{\left({}^{w}\;x_{t_{i}}\;-\;{}^{w}\;x_{b}\right)}^{2}+{\left({}^{w}\;y_{t_{i}}\;-\;{}^{w}\;y_{b}\right)}^{2}}{h} \end{array}\right]$$

where $a_{i}:=\left(f_{i}-r_{\lim}\;h_{\min}\right)$.

Considering the gradient of the complete index, all of these terms are added together, resulting:

$$\nabla J^{\prime }=-\epsilon_{d}\;\sum_{i=1}^{n}\frac{\left({}^{w}\;p_{t_{i}}\;-\;{}^{w}\;p_{b}\right)}{{\left(f_{i}-r_{\lim}\;h_{\min}\right)}^{2}}\;-\;\frac{\epsilon_{\gamma }}{h^{2}}\;\left[\begin{array}{c} \sum_{i=1}^{n}\;\;{}^{w}\;x_{t_{i}}\;-n\;{}^{w}\;x_{b}\\ \sum_{i=1}^{n}\;\;{}^{w}\;y_{t_{i}}\;-n\;{}^{w}\;y_{b}\\ \frac{\sum_{i=1}^{n}\left[{\left({}^{w}\;x_{t_{i}}\;-\;{}^{w}\;x_{b}\right)}^{2}+{\left({}^{w}\;y_{t_{i}}\;-\;{}^{w}\;y_{b}\right)}^{2}\right]}{h} \end{array}\right]$$

By expanding all of the terms by the components, we obtain:

$$\nabla J^{\prime }=\left[\begin{array}{c} -\sum_{i=1}^{n}\left(\frac{\epsilon_{d}}{{\left(f_{i}-r_{\lim}\;h_{\min}\right)}^{2}}+\frac{\epsilon_{\gamma }}{h^{2}}\right){}^{w}\;x_{t_{i}}\;+\;\left(\sum_{i=1}^{n}\frac{\epsilon_{d}}{{\left(f_{i}-r_{\lim}\;h_{\min}\right)}^{2}}+\frac{n\;\epsilon_{\gamma }}{h^{2}}\right){}^{w}\;x_{b}\\ -\sum_{i=1}^{n}\left(\frac{\epsilon_{d}}{{\left(f_{i}-r_{\lim}\;h_{\min}\right)}^{2}}+\frac{\epsilon_{\gamma }}{h^{2}}\right){}^{w}\;y_{t_{i}}\;+\;\left(\sum_{i=1}^{n}\frac{\epsilon_{d}}{{\left(f_{i}-r_{\lim}\;h_{\min}\right)}^{2}}+\frac{n\;\epsilon_{\gamma }}{h^{2}}\right){}^{w}\;y_{b}\\ h\;\epsilon_{d}\;\sum_{i=1}^{n}\frac{1}{{\left(f_{i}-r_{\lim}\;h_{\min}\right)}^{2}}-\frac{\epsilon_{\gamma }}{h^{3}}\sum_{i=1}^{n}\left[{\left({}^{w}\;x_{t_{i}}\;-\;{}^{w}\;x_{b}\right)}^{2}+{\left({}^{w}\;y_{t_{i}}\;-\;{}^{w}\;y_{b}\right)}^{2}\right] \end{array}\right]$$

Finding the solution for which the three gradient components become null gives rise to a set of three equations in the unknowns $\left\{{}^{w}\;x_{b},\;{}^{w}\;y_{b},\;{}^{w}\;z_{b}\right\}$, being ${}^{w}\;z_{b}\equiv h$:

(A5) $${}^{w}\;x_{b}=\frac{\sum_{i=1}^{n}\;\left(\frac{\epsilon_{d}}{{\left(f_{i}-r_{\lim}\;h_{\min}\right)}^{2}}\;+\;\frac{\epsilon_{\gamma }}{{{}^{w}\;z_{b}}^{2}}\right)\;{}^{w}\;x_{t_{i}}}{\sum_{i=1}^{n}\;\frac{\epsilon_{d}}{{\left(f_{i}-r_{\lim}\;h_{\min}\right)}^{2}}\;+\;\frac{\epsilon_{\gamma }}{{{}^{w}\;z_{b}}^{2}}\;n}$$

(A6) $${}^{w}\;y_{b}=\frac{\sum_{i=1}^{n}\;\left(\frac{\epsilon_{d}}{{\left(f_{i}-r_{\lim}\;h_{\min}\right)}^{2}}\;+\;\frac{\epsilon_{\gamma }}{{{}^{w}\;z_{b}}^{2}}\right)\;{}^{w}\;y_{t_{i}}}{\sum_{i=1}^{n}\;\frac{\epsilon_{d}}{{\left(f_{i}-r_{\lim}\;h_{\min}\right)}^{2}}\;+\;\frac{\epsilon_{\gamma }}{{{}^{w}\;z_{b}}^{2}}\;n}$$

(A7) $$\epsilon_{\gamma }{}^{w}\;\nu_{b}\sum_{i=1}^{n}\;\frac{\epsilon_{d}}{{\left(f_{i}-r_{\lim}\;h_{\min}\right)}^{2}}={{}^{w}\;\nu_{b}}^{2}\sum_{i=1}^{n}\left[{\left({}^{w}\;x_{t_{i}}\;-\;{}^{w}\;x_{b}\right)}^{2}+{\left({}^{w}\;y_{t_{i}}\;-\;{}^{w}\;y_{b}\right)}^{2}\right]$$

where ${}^{w}\;\nu_{b}$ is an intermediary variable ${}^{w}\;\nu_{b}:=\frac{\epsilon_{\gamma }}{{{}^{w}\;z_{b}}^{2}}$. The first two equations are a weighted average of the *x* and *y* components of the target locations. This set of equation has an analytical solution, which can be obtained by replacing the two first equations into the third one, in order to compute ${{}^{w}\;z_{b}}^{2}$ in the first place. This allows to transform Equation (A7) into a fourth order polynomial equation with the following form:

$$a_{4}\;{}^{w}\;\nu_{b}+a_{3}\;{}^{w}\;\nu_{b}+a_{2}\;{}^{w}\;\nu_{b}+a_{1}\;{}^{w}\;\nu_{b}+a_{0}=0$$

$$\begin{array}{ccc} a_{4} & = & n^{2}\sum \;(x_{t_{i}}^{2}\;+\;y_{t_{i}}^{2})-n{\left(\sum \;x_{t_{i}}\right)}^{2}\;-n{\left(\sum y_{t_{i}}\right)}^{2}\\ a_{3} & = & 2\;\left(\sum \;c_{i}\right)\;\left[n\;\sum \;(x_{t_{i}}^{2}+y_{t_{i}}^{2})-{\left(\sum \;x_{t_{i}}\right)}^{2}-{\left(\sum y_{t_{i}}\right)}^{2}\right]\\ a_{2} & = & -n^{2}\;\epsilon_{\gamma }\;\left(\sum \;c_{i}\right)+n\;{\left(\sum \;c_{i}\;x_{t_{i}}\right)}^{2}+n\;{\left(\sum \;c_{i}\;y_{t_{i}}\right)}^{2}+{\left(\sum \;c_{i}\right)}^{2}\;\sum \;(x_{t_{i}}^{2}\;+\;y_{t_{i}}^{2})-\\ & & -2\;\left(\sum c_{i}\right)\;\left[\left(\sum \;c_{i}\;x_{t_{i}}\right)\;\sum x_{t_{i}}+\left(\sum \;c_{i}\;y_{t_{i}}\right)\;\sum y_{t_{i}}\right]\\ a_{1} & = & -2\;n\;\epsilon_{\gamma }\;{\left(\sum \;c_{i}\right)}^{2}\\ a_{0} & = & -\epsilon_{\gamma }\;{\left(\sum \;c_{i}\right)}^{3} \end{array}$$

with:

$$c_{i}=\frac{\epsilon_{d}}{{\left(f_{i}-r_{\lim}\;h_{\min}\right)}^{2}}$$

This equation has only one real positive solution, providing the correct value for ${}^{w}\;\nu_{b}$. After this, ${}^{w}\;z_{b}$ is obtained:

$${}^{w}\;z_{b}=\sqrt{\frac{\epsilon_{\gamma }}{{}^{w}\;\nu_{b}}}$$

Then, by substituting ${}^{w}\;z_{b}$ in Equations (A5) and (A6), the other two components of the vehicle’s position are obtained.

Expressions (A5)–(A7) show that the first two components of the solution are a particular weighted average of the corresponding coordinates of the target locations. This average, however, is not immediate, since it depends on the solution value of the altitude. If we give the name $\mu^{•}$ to this particular average of the target locations on the ground plane, $\mu^{•}:={\left[{}^{w}\;x_{b},\;{}^{w}\;y_{b},\;0\right]}^{\;⊤}$, we can see that Equation (A7) can be rewritten as:

$${}^{w}\;z_{b}={\left[\frac{\epsilon_{\gamma }\;n}{\epsilon_{d}\sum_{i=1}^{n}\;\frac{1}{{\left(f_{i}-r_{\lim}\;h_{\min}\right)}^{2}}}\;\mathrm{tr}\left(V^{•}\right)\right]}^{\frac{1}{4}}$$

where $V^{•}$ is the covariance matrix of the set of target positions, with respect to the defined average $\mu^{•}$:

$$V^{•}=\frac{1}{n}\;\sum_{i=1}^{n}\left({}^{w}\;p_{t_{i}}\;-\;\mu^{•}\right){\left({}^{w}\;p_{t_{i}}\;-\;\mu^{•}\right)}^{\;⊤}$$

Of course, these relations do not avoid the need to solve the set of equations in its original form, but they provide significant insight into the solutions provided by the approximate uniform optimization index. In particular, in relation to the third component, it is now clear that it essentially depends on the dispersion or scattering of the targets on the ground plane, with respect to a particular ”central” point defined by $\mu^{•}$. The more scattered the targets are, the higher the altitude is required; verticality is thus favored.

There is a particular case of a system of Equations (A5)–(A7) that deserves further discussion. That is when all of the tracking cameras have the same focal length: $f_{i}\;=\;f$, ∀i=1…n. It is interesting to verify that, under this condition, Equations (A5) and (A6) reduce to the following:

$${}^{w}\;x_{b}=\mu_{x}=\frac{1}{n}\sum_{i=1}^{n}\;{}^{w}\;x_{t_{i}}$$

$${}^{w}\;y_{b}=\mu_{y}=\frac{1}{n}\sum_{i=1}^{n}\;{}^{w}\;y_{t_{i}}$$

That is, as far as the first two components are concerned, the optimal solution is to take the plain average of the target positions: $\mu ={\left[\mu_{x},\;\mu_{y},\;0\right]}^{\;⊤}$:

$$\mu =\frac{1}{n}\;\sum_{i=1}^{n}{}^{w}\;p_{t_{i}}$$

This is justified by the fact that, under these circumstances, optical range is equivalent to Euclidean distance. With regard to the third component, assuming that the first two coordinates take the stated values, the solution adopts the form:

$${}^{w}\;z_{b}={\left[\epsilon_{\gamma }\;{\left(\frac{f}{r_{\lim}}\;-\;\;h_{\min}\right)}^{2}\;\mathrm{tr}\left(V\right)\right]}^{\frac{1}{4}}$$

where V is the conventional covariance matrix of the set of target positions:

$$V=\frac{1}{n}\;\sum_{i=1}^{n}\left({}^{w}\;p_{t_{i}}\;-\;\mu \right){\left({}^{w}\;p_{t_{i}}\;-\;\mu \right)}^{\;⊤}$$

The convexity analysis of this simplified index is not required, since the uniqueness of the optimal solution is an immediate consequence of the provided analytical inference.
